# Cholesteryl α-D-glucoside 6-acyltransferase enhances the adhesion of *Helicobacter pylori* to gastric epithelium

**DOI:** 10.1038/s42003-020-0855-y

**Published:** 2020-03-13

**Authors:** Hau-Ming Jan, Yi-Chi Chen, Tsai-Chen Yang, Lih-Lih Ong, Chia-Chen Chang, Sasikala Muthusamy, Andualem Bahiru Abera, Ming-Shiang Wu, Jacquelyn Gervay-Hague, Kwok-Kong Tony Mong, Chun-Hung Lin

**Affiliations:** 1 Institute of Biological Chemistry, Academia Sinica, No. 128 Academic Road Section 2, Nan-Kang, Taipei, 11529 Taiwan; 2 Department of Chemistry and Institute of Biochemical Sciences, National Taiwan University, Taipei, 10617 Taiwan; 3 Department of Applied Chemistry, National Chiao Tung University, Hsin-Chu, 30010 Taiwan; 4 Sustainable Chemical Science and Technology, Taiwan International Graduate Program, Academia Sinica and National Chiao Tung University, Taipei, 11529 Taiwan; 5 Molecular and Biological Agricultural Sciences, Taiwan International Graduate Program, Academia Sinica and National Chung-Hsing University, Taipei, 11529 Taiwan; 6 Graduate Institute of Biotechnology, National Chung-Hsing University, Taichung, 40227 Taiwan; 7 Division of Gastroenterology, Department of Internal Medicine, National Taiwan University Hospital, Taipei, 10002 Taiwan; 8 Department of Chemistry, University of California, Davis, CA 95618 USA

**Keywords:** Cellular microbiology, Integrins, Pathogens

## Abstract

*Helicobacter pylori*, the most common etiologic agent of gastric diseases including gastric cancer, is auxotrophic for cholesterol and has to hijack it from gastric epithelia. Upon uptake, the bacteria convert cholesterol to cholesteryl 6′-*O*-acyl-α-D-glucopyranoside (CAG) to promote lipid raft clustering in the host cell membranes. However, how CAG appears in the host to exert the pathogenesis still remains ambiguous. Herein we identified *hp0499* to be the gene of cholesteryl α-D-glucopyranoside acyltransferase (CGAT). Together with cholesteryl glucosyltransferase (catalyzing the prior step), CGAT is secreted via outer membrane vesicles to the host cells for direct synthesis of CAG. This significantly enhances lipid rafts clustering, gathers adhesion molecules (including Lewis antigens and integrins α5, β1), and promotes more bacterial adhesion. Furthermore, the clinically used drug amiodarone was shown as a potent inhibitor of CGAT to effectively reduce the bacterial adhesion, indicating that CGAT is a potential target of therapeutic intervention.

## Abstract (web summary)

Jan et al. identify cholesteryl α-D- glucopyranoside acyltransferase as a key enzyme in *Helicobacter pylori*’s synthesis of cholesteryl 6’-O-acyl-α-D-glucopyranoside, which promotes bacterial adhesion. This study provides insights into the *H. pylori*-induced pathogenesis and therapeutic strategies against it.

## Introduction

*Helicobacter pylori* infects more than half of the world’s population^1^. The bacterial infection not only results in various gastrointestinal diseases that include gastric carcinoma and gastric mucosa-associated lymphoid tissue lymphoma, but also represents a leading cause of cancer-related deaths^2^. The pathogenicity of *H. pylori* is closely associated with the genes of *cag*-pathogenicity island, the 40-kb-long sequence of 31 coding regions including the type IV secretion system (T4SS)^3,4^. Following the attachment of *H. pylori* to gastric epithelial cells, the T4SS apparatus injects the *cag*-pathogenicity island-encoded protein CagA into the attached host cells. Once delivered inside the cells, CagA undergoes tyrosine phosphorylation by c-Src family kinases. The phosphorylated CagA then activates several cellular signaling processes, including those leading to cancer development^5^.

The attachment of *H. pylori* to gastric epithelia is a necessary process for colonization, as well as an initial step in the pathogenesis^6^. The increasing level of *H. pylori* adhesion was found relevant to several deteriorating developments, such as epithelial cell degeneration and mucin depletion. Among several important factors contributing to the bacterial adhesion, BabA is the best characterized adhesin that recognizes Lewis^b^/ABO blood group antigens^7,8^. Another adhesin SabA binds specifically to sialyl Lewis^x^ and sialyl Lewis^a^ antigens^9^. The T4SS pili of *H. pylori*, probably assembled upon an initial contact with host cells^10^, are known to interact with the integrins α5 and β1 to enhance the adherence^11,12^. Furthermore, the bacterium produces cholesteryl α-glucoside derivatives to affect the bacterial adhesion^13^.

*H. pylori* is auxotrophic for cholesterol. It assimilates cholesterol into its membrane by taking up cholesterol from epithelial cells of the stomach. Upon uptake, the bacterial cells modify the cholesterol by α-glucosylation. Specifically, the glucosyltransferase encoded by *hp0421* catalyzes the transfer of glucose to the 3-hydroxyl group of cholesterol, yielding cholesteryl α-d-glucopyranoside (CG). There is a subsequent modification occurring at O6′ of glucose in CG, i.e., cholesteryl 6′-*O*-acyl-α-d-glucopyranoside (CAG) or cholesteryl 6′-*O*-phosphatidyl-α-d-glucopyranoside are made by attaching an acyl or phosphatidyl group, respectively. We previously developed a metabolite-tagging method for characterizing these derivatives with a *femto*-molar detection limit^14^. The subsequent analysis led to the findings that these bacteria acquire phospholipids from the membrane of epithelial cells for CAG biosynthesis. The increase in longer CAG acyl chains helped to promote lipid raft clustering in the host cell membranes and thus the delivery of virulence factor CagA into the host cell^14^.

Herein we report the identification of *hp0499* as the gene of cholesteryl α-d-glucopyranoside 6′-acyltransferase (CGAT), as well as characterization of the corresponding recombinant protein. The enzyme is located in the outer membrane of *H. pylori*, secreted extracellularly in the form of outer membrane vesicles (OMVs), and thus delivered to the host cells to directly produce CAGs there for the lipid raft-mediated enhancement of *H. pylori* adhesion. Additionally, a potent CGAT inhibitor was discovered to effectively blockade the *H. pylori* adhesion, demonstrating CGAT to be a potential target of therapeutic intervention.

## Results

### Acyl chain length of CAG affects bacterial adhesion

**Fig. 1:**
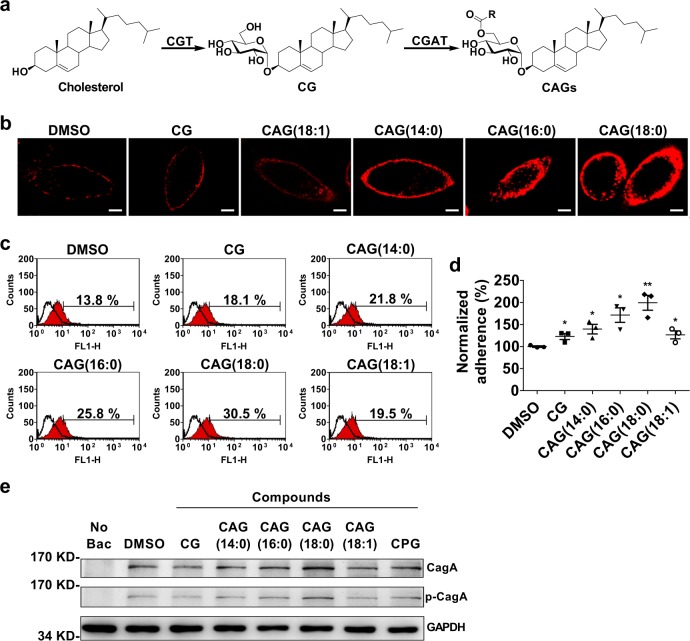
CAGs of varied chain length were able to enhance *H. pylori* adhesion and the corresponding CagA translocation. **a** Biosynthetic pathway of CAG in all *H. pylori* strains where cholesterol α-glucosyltransferase (CGT) and cholesteryl α-d-glucoside acyltransferase (CGAT) consecutively catalyze the reactions to yield cholesteryl α-d-glucopyranoside (CG) and CAG, respectively. The R group of CAG represents O6′-esters of different fatty acids, e.g., myristic acid (14:0), palmitic acid (16:0), stearic acid (18:0), and oleic acid (18:1). **b** Representative confocal images of lipid rafts clustering in the presence of CG or CAGs with different acyl chain. After AGS cells were treated with CG or CAG (as indicated) for 1 h, the lipid rafts (GM1) were then labeled with Alexa Fluor 594-conjugated cholera toxin subunit b (red fluorescence). Confocal images were collected under a Leica SP5 X inverted confocal microscope. Scale bar: 5 μm. **c**, **d** Degree of *H. pylori* adherence to AGS cells is dependent on the acyl chain of CAG. AGS cells were first treated with CG or CAGs (as indicated) for 1 h, infected with *H. pylori* strain 26695 for another 1 h, and then subjected to flow cytometry analysis. Adherence was measured as the proportion of adhered AGS cells with *H. pylori* (%; shown in each plot). The resulting quantitation of cell adherence was normalized in comparison with the control and shown in **d**. Data are shown as mean ± SD (standard deviation). All statistically significant differences are indicated with asterisks; **p < 0.01, *p < 0.05 vs. the control (*n* = 3). **e** Effects of CAGs with different acyl chains on CagA translocation and CagA tyrosine phosphorylation that was detected by immunoblotting after the co-culture of AGS cells with *H. pylori* 26695 as described in (**c**). Data sources for **d** are provided in Supplementary Data 1. Uncropped immunoblot images for **e** are provided in Supplementary Fig. 4.

Figure 1a shows the biosynthetic pathway of cholesterol-α-glucosides. Upon uptake of cholesterol, *H. pylori* employs cholesterol glucosyltransferase (CGT) to convert cholesterol to CG, followed by the reaction of CGAT to catalyze the acyltransfer to produce CAG. We previously demonstrated that CAG, rather than CG or cholesteryl 6′-*O*-phosphatidyl-α-d-glucopyranoside, was able to promote lipid rafts clustering in the host cell membranes, as well as CagA translocation and phosphorylation^14^. Wang et al.^13^ reported that the loss of cholesteryl-α-glucoside derivatives abolished the adhesion of *H. pylori* to AGS cells^13^. Both studies provide the impetus to understand if CAG is the key to regulate the bacterial adhesion. Among CG and CAGs of different chain length (such as CAG(14:0), CAG(16:0), CAG(18:0), and CAG(18:1)) added to the culture of AGS cells, CAG(18:0) enhanced the lipid rafts clustering to the highest degree when ganglioside GM1 was utilized to label the formation of lipid rafts (Fig. 1b). Furthermore, AGS cells were treated with each of these CG and CAGs, infected with *H. pylori* 26695 and then examined for the extent of adhesion by flow cytometry. The result was consistent with that obtained from the lipid rafts study, i.e., the longer the acyl chain was, the higher levels there were in the bacterial adhesion (Fig. 1c, d), CagA translocation, and the corresponding tyrosine phosphorylation (Fig. 1e). Interestingly, these studies were not favored by unsaturation in the acyl chain, suggesting that the membrane fluidity or packing in the lipid chains appears to be critical.

### Identification and characterization of *H. pylori* CGAT

Previously phospholipids were shown to serve as the precursors for the biosynthesis of CAG and cholesteryl 6′-*O*-phosphatidyl-α-d-glucopyranoside^14^. In an attempt of identifying the enzyme to catalyze the formation of CAGs, we performed several gene-knockout strains related to phospholipase, esterase, or acyltransferase (see Methods section for the list of candidates) by inserting a kanamycin-resistance cassette into desirable genes of *H. pylori* 26695. Among these gene-knockout strains, only *Δhp0499* did not produce a detectable level of CAGs, suggesting that the *Δhp0499* strain is a CGAT-deficient strain (ΔCGAT) (Supplementary Fig. 1a). We additionally prepared the CGAT-knock-in strain by inserting the intact *hp0499* gene with a chloramphenicol-resistance cassette to the genome upstream of the aforementioned *hp0499*-modified sequence. The CGAT-knock-in strain indeed restored the capability of synthesizing CAG (Supplementary Fig. 1a). Both data confirmed that *hp0499* is the gene responsible for CAG production.

Two recombinant forms of CGAT were overexpressed in *Escherichia coli*, including one containing an N-terminal 6x His tag and the other having an N-terminal maltose-binding protein (MBP-CGAT). Despite several problems initially encountered, such as low expression level, formation of inclusion bodies and protein instability, we finally overcame these difficulties after numerous trials and errors. MBP-CGAT displayed fourfold higher activity than the other His-tagged CGAT (Supplementary Fig. 1b) by using LC-MS analysis to measure CAG(14:0) that resulted from the enzymatic reaction of CG and 1,2-dimyristoyl-sn-glycero-3-phosphatidylethanolamine (PE(14:0,14:0)). As a consequence, only MBP-CGAT (hereafter called CGAT) was characterized in the following studies. The enzyme has two substrates, namely phosphatidylethanolamine (PE) as the acyl donor and CG as the acceptor. Since PE contains two acyl chains, we thus utilized PE(d16:0/18:1) (i.e., deuterium-labeled palmitic acid (16: 0) and unlabeled oleic acid (18:1) at a1- and a2-positions of the glycerol moiety, respectively) to identify which acyl chain in the PE was hydrolyzed and transferred to CG. The resulting analysis indicated that CGAT belongs to the family of phospholipase A1 to catalyze the formation of CAG(d16:0) and 1-hydroxy-2-oleoyl-sn-glycero-3-phosphoethanolamine (lyso-PE(18:1)), as shown in Supplementary Fig. 1c, d, respectively. Furthermore, CGAT was active in the range of pH 3.5–9.0 with an optimum activity at pH 4.5 (Supplementary Fig. 1e). Lineweaver–Burk plot analysis of CGAT steady-state kinetics indicated that the catalysis operates through a sequential rapid-equilibrium random bi-bi mechanism (Supplementary Fig. 1f, g). The values of *K*_CG_ and *K*_PE_ were 26.0 and 290 μM, respectively, and the turnover number was close to one per sec (*k*_cat_ = 58.5 min^−1^).

Meanwhile, we also examined the metal ion dependence and substrate specificity on phospholipids. Among seven divalent metal ions, the enzyme became the most active in the presence of calcium ion (Supplementary Fig. 1h). CGAT displayed broad specificity for several lipids (Supplementary Fig. 1i, j); in particular, a phosphatidyl moiety is required for the recognition. Acceptable substrates need to have two acyl chains (see the rate difference between PE vs. 1-myristoyl-2-hydroxy-sn-glycero-3-phosphoethanolamine (2-lyso-PE) although they both can be saturated (Supplementary Fig. 1i) or the one at a2-position is unsaturated (Supplementary Fig. 1j). Obviously, CGAT mainly preferred PE (100% relative activity), phosphatidylserine (PS, ~25–50% activity depending on the lipid compositions) and phosphatidylcholine (PC, ~4–17% activity). In consistence with the previous result that CAGs of varied chain length existed in the co-culturing of AGS cells with *H. pylori*, CGAT accepted all the five phosphatidylcholines of different chain lengths (Supplementary Fig. 1k). We did not examined PEs simply because most PEs are rarely soluble in the assay buffer (100 mM sodium formate, 25 mM CaCl_2,_ pH 4.5).

### Cellular locations of CGAT

**Fig. 2:**
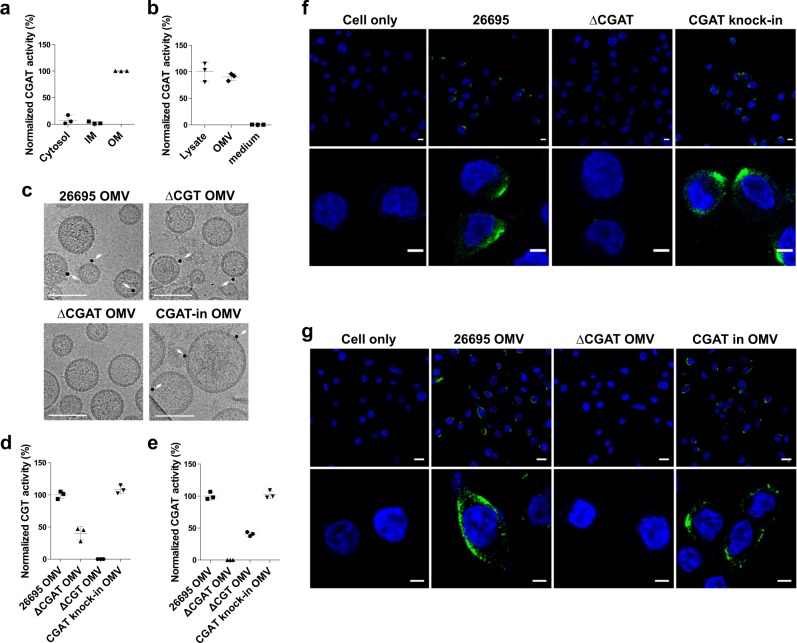
Cellular location of CGAT. **a, b** Cellular location of CGAT was determined by the enzyme activity. **a** IM and OM stand for the abbreviations of inner and outer membranes, respectively. See Methods section for the procedure to obtain the fractions of cytosol, IM, and OM of *H. pylori* 26695. **b** The three fractions were obtained from the same cell number of *H. pylori* 26695. These fractions were individually incubated for 2 h with the mixture of CG and PE at pH 4.5. The resulting CAG products were analyzed by LC-MS analysis. Data were obtained from three replicates. Data are shown as mean ± SD. **c** CGAT existing in OMVs was identified by cryo-transmission electron microscopy. OMVs were purified from different *H. pylori* strains and examined if they contained CGAT that is labeled by immune-gold (shown in black dots and indicated by white arrows). Scale bar: 50 nm. **d**, **e** Levels of CGT (**d**) and CGAT (**e**) activities in the OMVs that were purified from different *H. pylori* strains and determined by LC-MS analysis. Data are shown as mean ± SD. **f** CGAT was shown in the co-culture of AGS cells with different *H. pylori* strains. After co-culture with *H. pylori* for 8 h, the cells were fixed and stained with CGAT-specific antibody (Alexa Fluor 488, green). Nuclei were counterstained with DAPI (blue). Lower panels showed the magnified images from a part of upper panels. Scale bars denoted 20 (upper) and 5 (lower) μm. **g** CGAT was shown in the AGS cells with a prior treatment with or without *H. pylori* 26695 OMV, ΔCGAT OMV, or CGAT-knock-in OMV for 8 h. The cells were then stained as described in (**f**). Scale bars denote 20 (upper) and 5 (lower) μm. Data sources for **a**, **b**, **d**, and **e** are provided in Supplementary Data 1.

To study the cellular location of CGAT, the cells of *H. pylori* 26695 were cultured, lysed, and then extracted by *N*-lauroylsarcosine and n-dodecyl-β-d-maltopyranoside to obtain the fractions of cytosol, inner and outer membranes^15^. Enzyme activity assay was conducted to determine where CGAT is localized. The enzyme was found to exist mainly in the outer membrane, but not in the other locations (Fig. 2a), which is coherent to the proposed idea by Scott et al.^16^ that most *H. pylori* surface proteins have high values of isoelectric point to likely resist the acidic environment. The isoelectric point of CGAT is 9.26. Moreover, the CGAT activity was observed in the OMVs, rather than in the culture medium (Fig. 2b), suggesting that CGAT resided in the outer membrane can be enclosed in the OMVs and secreted to the extracellular space. Figure 2c showed the direct evidence from cryo-transmission electron microscopy that CGAT was present in the OMVs isolated from the strains of *H. pylori* 26695, ΔCGT-knock-out, and CGAT-knock-in, instead of ΔCGAT-knock-out. When these OMVs were isolated from *hp0499*-containing strains and then surveyed for proteomic analysis, CGAT was shown among a range of 310–420 proteins identified (Supplementary Table 1). Further activity analysis of these OMVs (Fig. 2d, e) concluded that *H. pylori* OMVs contained the two enzymes CGT and CGAT. As shown by confocal fluorescence microscopy (Fig. 2f, g, Supplementary Movie 1), CGAT was translocated to AGS cells after the cells had been pretreated with *H. pylori* 26695 OMVs or infected by *H. pylori* 26695.

### CGAT is delivered to host cell to enhance *H. pylori* adhesion

**Fig. 3:**
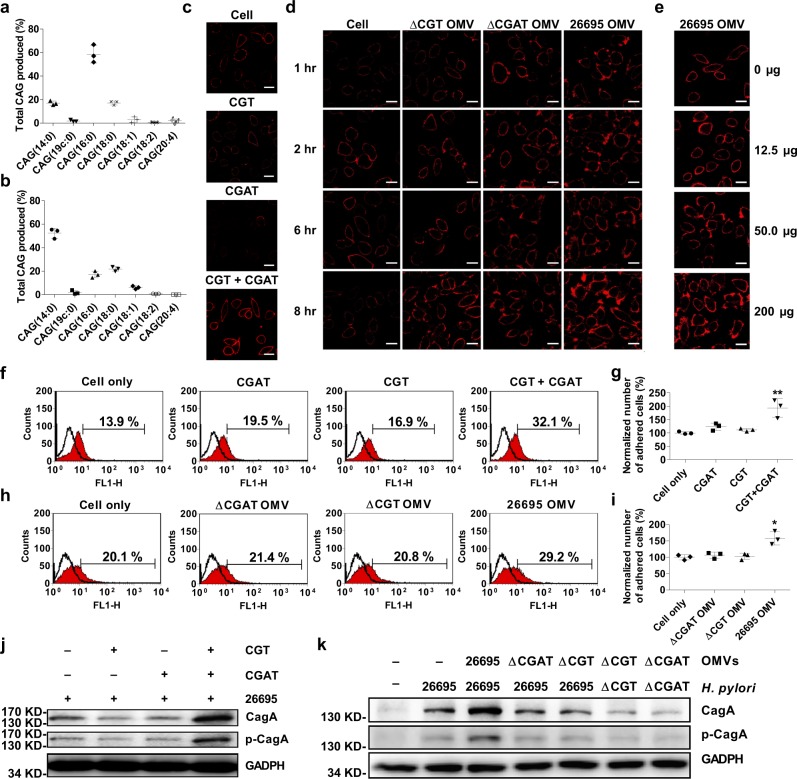
Enhanced levels in lipid rafts clustering, adhesion, and CagA translocation by the concerted actions of CGAT and CGT. **a**, **b** The compositions of CAGs in AGS cells after the cells were treated with both recombinant CGT and CGAT (**a**), or with the OMVs of *H. pylori* 26695 (**b**). **c** Enhanced level of lipid raft clustering was observed after AGS cells were treated with both recombinant CGT and CGAT for 8 h. Red fluorescence denotes the lipid rafts. Scale bar: 20 μm. **d**, **e** Time- and does-dependent confocal images of lipid raft clustering in AGS cells. **d** The images were obtained after the cells were treated with the purified OMVs (200 μg) from different *H. pylori* strains at 1, 2, 6, and 8 h. **e** The images were generated after the cells were treated with *H. pylori* 26695 OMVs for 8 h. Red fluorescence denotes the lipid rafts. Scale bar: 20 μm. **f**, **h** Enhanced *H. pylori* adherence to AGS cells after the cells were incubated with recombinant CGT or/and CGAT (**f**) or with the purified OMVs from different *H. pylori* strains (**h**). AGS cells were treated with the enzymes or OMVs for 8 h and then infected with *H. pylori* 26695 for another 1 h. Adherence was examined by flow cytometry analysis and shown as the proportion of adhered cells with *H. pylori* (%; shown in each plot). **g**, **i** Quantitation of the adhered cells of **f** and **h** was normalized in comparison with the negative control, respectively. All statistically significant differences are indicated with asterisks; **p < 0.01, *p < 0.05 vs. control group (*n* = 3). **j**, **k** Immunobloting analysis on CagA translocation and the related tyrosine phosphorylation to examine the effect of recombinant enzymes (**j**) or purified OMVs (**k**). The procedure was similar to that of **f** and **h**. The uncropped images are provided in Supplementary Fig. 4. In **a**, **b**, **g** and **i**, data are shown as mean ± SD and the data sources are provided in Supplementary Data 1.

It is known that CAG(14:0) and CAG(19c:0) mainly exist in *H. pylori*, as the reaction products of CGAT. To understand how the translocated CGAT possibly functions in the host cell, the primary concern is to analyze the CGAT products once the enzyme becomes available in the host cells. We treated AGS cells with recombinant proteins CGT and CGAT both to mimic the circumstances of bacterial infection. There was a significant change in the CAG compositions (Fig. 3a), i.e., most CAGs contain longer fatty acid chains, including (16:0), (18:0), and (18:1), after the treatment of AGS cells with both CGT and CGAT. Apparently the translocated CGAT accepted available PEs or phosphatidylserines in AGS cells. Likewise, the treatment with *H. pylori* 26696 OMVs also resulted in a similar profile (Fig. 3b). In agreement with the aforementioned observation that CGT and CGAT were delivered to the host cells by the OMVs (Fig. 2f, g), Fig. 3b indicated that the two enzymes, once delivered to the host cells, became functional to synthesize CAGs of longer acyl chains. The result is coherent to the previous report^14^ that the co-culture of *H. pylori* with AGS cells generated a significant amount of CAGs containing longer or/and unsaturated acyl chains.

The aforementioned enzyme or OMV treatment also enhanced the degree of lipid rafts clustering in a time- (Fig. 3c, d) and dose-dependent manner (Fig. 3e). Furthermore, we had two observations to corroborate the indispensable production of CAGs. There was no enhancement of lipid rafts clustering if only CGT or CGAT was present in the study. Lipid rafts clustering only became evident in the regions to which *H. pylori* 26695 OMVs were attached, as shown by the real-time imaging (Supplementary Movie 1), in contrast to those without the OMV attachment. Additional examinations on the effect of *H. pylori* adhesion also showed the consistent trend, such as flow cytometry analysis (Fig. 3f–i), cagA translocation, and the corresponding tyrosine phosphorylation (Fig. 3j, k). Taken together, these results indicated that the presence of both CGT and CAGT in the host cells is the prerequisite for the enhancement of lipid raft clustering, bacterial adhesion, and the subsequent virulence.

### CAGs help to gather adhesion molecules

**Fig. 4:**
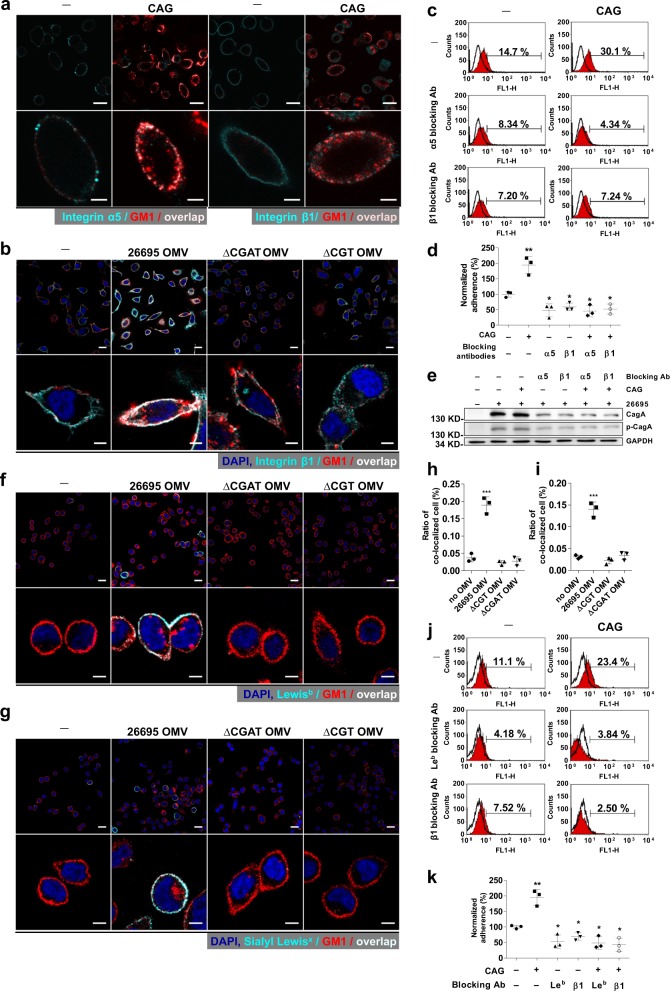
Gathering of integrins α5/β1, Lewis^b^, and sialyl Lewis^x^ by CAG or CAG-containing OMV in the rafts region. **a** Integrins α5 and β1 and **b** Integrin β1 gathered at the rafts region by treatment of AGS cells with CAG (**a**) or with *H. pylori* 26695 OMVs (**b**). AGS cells were treated with CAG(18:0) for 1 h (**a**) or with OMVs for 8 h (**b**), and then fixed. Immunofluorescent staining was then applied to indicate lipid rafts (red), integrin α5 or β1 (cyan), and nuclei (blue). The co-localization of the first two is shown in color of fair pink. Scale bars: 20 (upper) and 5 (lower) μm. **c**–**e** Effects of integrin-blocking antibodies on *H. pylori* adhesion, CagA translocation, and the related tyrosine phosphorylation. AGS cells were treated with or without the integrin α5- or β1-blocking antibodies for 1 h, and incubated in the presence or absence of CAG(18:0) for additional 1 h, and then infected with *H. pylori* 26695. Adherence was measured by flow cytometry analysis (**c**) as the proportion of adhered cells with *H. pylori* (%; shown in each plot). The quantitation made in **c** was summarized in (**d**). Levels of CagA translocation and CagA tyrosine phosphorylation were detected by immunoblotting and shown in **e**. **f**, **g** Gathering of Lewis^b^ (**f**) and sialyl Lewis^x^ (**g**) in the rafts region by treatment of KATO III cells with *H. pylori* 26695 OMVs. The procedure and staining were similar to those of (**b**). Scale bars: 20 (upper) and 5 (lower) μm. **h** and **i** stand for quantitation of the confocal images made in **f** and **g**, respectively. **j**, **k** Effect of Lewis^b^-blocking antibodies on *H. pylori* adhesion. The procedure was similar to that of **c** and **d**. In **d**, **h**, **i**, and **k**, data are provided in Supplementary Data 1 and shown as mean ± SD and all statistically significant differences are indicated with asterisks; ***p < 0.001, **p < 0.01, *p < 0.05 vs. the control group (*n* = 3). Uncropped immunoblot images for **e** are provided in Supplementary Fig. 4.

Although lipid rafts clustering and the chemoattraction of cholesterol^17^ is a driving force for *H. pylori* to move toward the rafts region, leading to the enhancement of bacterial adhesion, the detailed mechanism still remains unclear. It was reported that integrins α5 and β1 were recruited to the raft region when lipid rafts were clustered in T cells^18,19^. Both integrins were shown in gastric epithelial cells to interact with T4SS of *H. pylori* to enhance the bacterial adhesion^11^. To examine a possible correlation of the integrins with the CAG-induced clustering of lipid rafts, we first found that integrins α5 and β1 were both co-localized with CAG-induced lipid rafts in AGS cells (Fig. 4a). *H. pylori* 26695 OMVs, rather than those containing CGT or CGAT only, were able to recruit integrin β1 to the raft regions in AGS and KATO III cells (Fig. 4b and Supplementary Fig. 2, respectively). Despite enhanced *H. pylori* adhesion to AGS cells in the presence of CAG(18:0), the adherence and the subsequent CagA translocation were inhibited by integrin α5- or β1-blocking antibodies (Fig. 4c–e).

Like the integrins, Lewis^b^ and sialyl Lewis^x^ antigens were able to congregate in the rafts region induced by the treatment of *H. pylori* 26695 OMVs in KATO III cells (that were used instead of AGS cells because the latter cells have extremely low expression of Lewis antigens, including Lewis^b^ and sialyl Lewis^x^). Figure 4f, h displayed the co-localization of the lipid rafts and Lewis^b^. Overlapped staining of the lipid rafts and sialyl Lewis^x^ was shown in Fig. 4g, i. Again in these figures, *H. pylori* 26695 OMVs produced higher co-localization signals than the OMVs isolated from the knockout strains. Likewise, the use of Lewis^b^- or integrin β1-blocking antibodies also significantly blockaded *H. pylori* adhesion to KATO III cells in the presence of CAG(18:0), as shown in Fig. 4j, k. These results suggested that CAG-induced clustering of lipid rafts gathered bacterial adhesion-related molecules in the raft regions, such as the integrins, Lewis^b^, and sialyl Lewis^x^, resulting in the enhancement of bacterial adhesion.

### Amiodarone is a CGAT inhibitor to prevent *H. pylori* adhesion

**Fig. 5:**
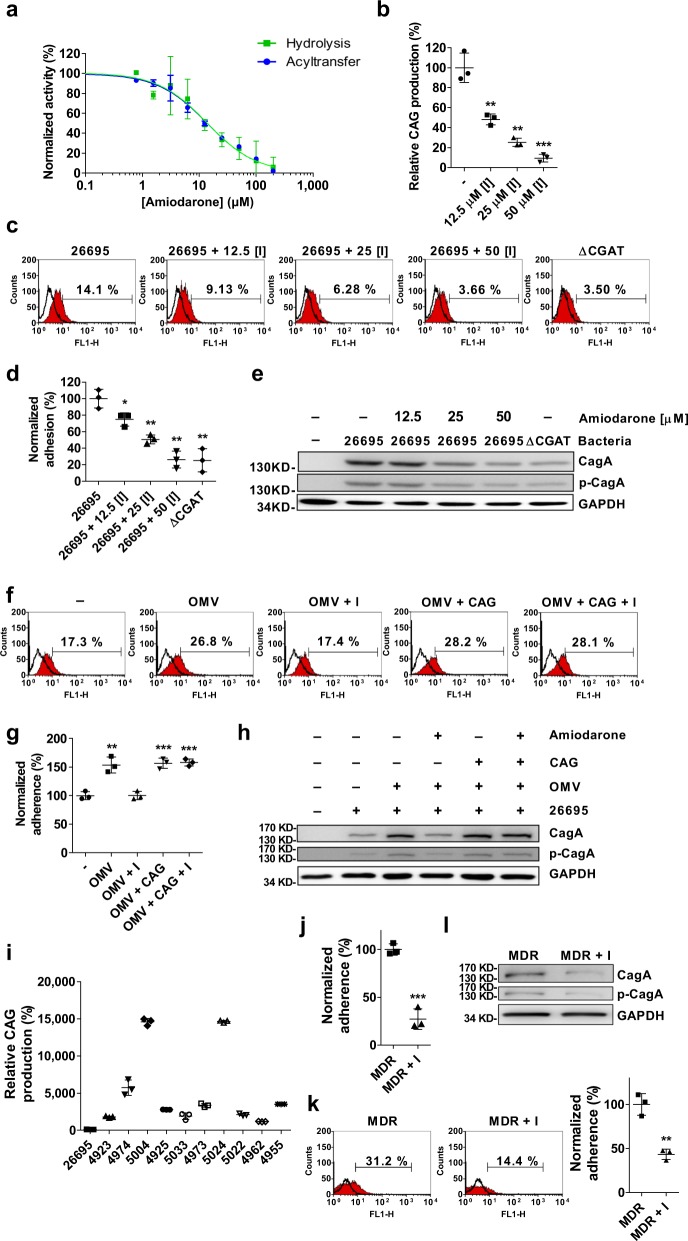
Amiodarone effectively prevented *H. pylori* from adhesion. **a** Concentration–response plots of the inhibition against the CGAT-catalyzed hydrolysis of PE (14:0,14:0) (green) and the corresponding acyltransfer (blue). **b** Effect of amiodarone on the CAG formation in *H. pylori*. *H. pylori* 26695 cells were treated with amiodarone for 2 days, followed by LC-MS analysis. **c**–**e** Effects of amiodarone on *H. pylori* adhesion (**c**, **d**), CagA translocation, and CagA tyrosine phosphorylation (**e**). After treatment with amiodadrone, *H. pylori* 26695 or the mutant strain ΔCGAT was cultured with AGS cells for 1 h. Degree of adhesion was measured by flow cytometry analysis (**c**) and quantitated as the proportion of adhered cells with *H. pylori* (%, shown in each plot). The quantitation was summarized in (**d**). **f**–**h** Effects of amiodarone and OMVs on *H. pylori* adhesion (**f**, **g**), CagA translocation, and CagA tyrosine phosphorylation (**h**). AGS cells were treated with or without amiodarone in the presence of *H. pylori* 26695 OMVs (or the OMVs that had been pretreated with CAG(18:0)) for 8 h and then infected with *H. pylori* 26695 for another 1 h. **f**, **g** Degree of adhesion was shown in the same manner as **c** and **d**, respectively. **i** CAG levels in *H. pylori* 26695 and ten MDR strains clinically isolated. The bacterial strains were cultured for 2 days, followed by LC-MS analysis. **j** Effect of amiodarone on the CAG formation (shown by LC-MS analysis) in MDR 5024 strain. **k**, **l** Effects of amiodarone on the adhesion of MDR 5024 (**k**), CagA translocation, and the tyrosine phosphorylation (**I**). After treatment with amiodarone, MDR 5024 infected AGS cells for 1 h. In **j**–**l**, MDR+I denoted the incubation of MDR 5024 with amiodarone. In **b**, **d**, **g**, **j** and **k**, all statistically significant differences are indicated with asterisks; ***p < 0.001, **p < 0.01, *p < 0.05 vs. the control group (*n* = 3). In **a**–**d**, **g**, **i**–**k**, data are shown as mean ± SD, and provided in Supplementary Data 1. Uncropped immunoblot images for **e**, **h**, and **l** are provided in Supplementary Fig. 4.

To study if the enzyme is a target of therapeutic intervention, we screened a number of molecules with the activity assay of CGAT and then identified amiodarone to be a potent inhibitor (IC_50_ = 13.8 μM, Fig. 5a). The compound was then added to *H. pylori*-infected AGS cells for further evaluation. As compared to the negative control, 50 μM amiodarone significantly reduced the production of CAG in AGS cells, bacterial adhesion, CagA translocation, and the related tyrosine phosphorylation (Fig. 5b–e, respectively). Apparently, inhibiting CGAT activity is the key to decrease the virulence of *H. pylori*. In the presence of OMVs, amiodarone still effectively abolished the adherence of *H. pylori* to AGS cells (Fig. 5f, g), as well as the related CagA translocation (Fig. 5h). Moreover, we also examined if amiodarone caused any effect other than CGAT inhibition. Before AGS cells were infected with *H. pylori*, the AGS cells were incubated with (or without) amiodarone and the OMVs that had been treated with CAG(18:0). The result showed no difference in the degree of bacterial infection, and the related CagA translocation (Fig. 5f–h) no matter if amiodarone was present. Meanwhile, we also obtained a similar result in the following study, i.e. no difference in the presence or absence of amiodarone. *H. pylori* was treated with (or without) amiodarone in the presence of CAG(18:0) before the bacteria infected AGS cells (Supplementary Fig. 3). These results suggested that amiodarone takes effect only on the CAG-induced changes via inhibition of CGAT activity.

Furthermore, we examined the CAG levels in ten multiple drug-resistant (MDR) strains of *H. pylori* that displayed resistance to the treatment of levofloxacin, metronidazole, and clarithromycin. Interestingly, their CAG levels were 10–150 times higher than that of *H. pylori* 26695 (Fig. 5i). To verify if amiodarone also inhibits the CGAT activity and the related pathogenesis in these resistant strains, one strain (5024) producing the highest CAG level had been incubated with amiodarone before it infected AGS cells. To our delight, at the concentration of 50 μM, aminodarone significantly reduced not only the CAG level (Fig. 5j) but also the bacterial adhesion (Fig. 5k), CagA translocation, and the related tyrosine phosphorylation (Fig. 5l). These results pave the way for further development of CGAT inhibitors for the purpose of therapeutic intervention.

**Fig. 6:**
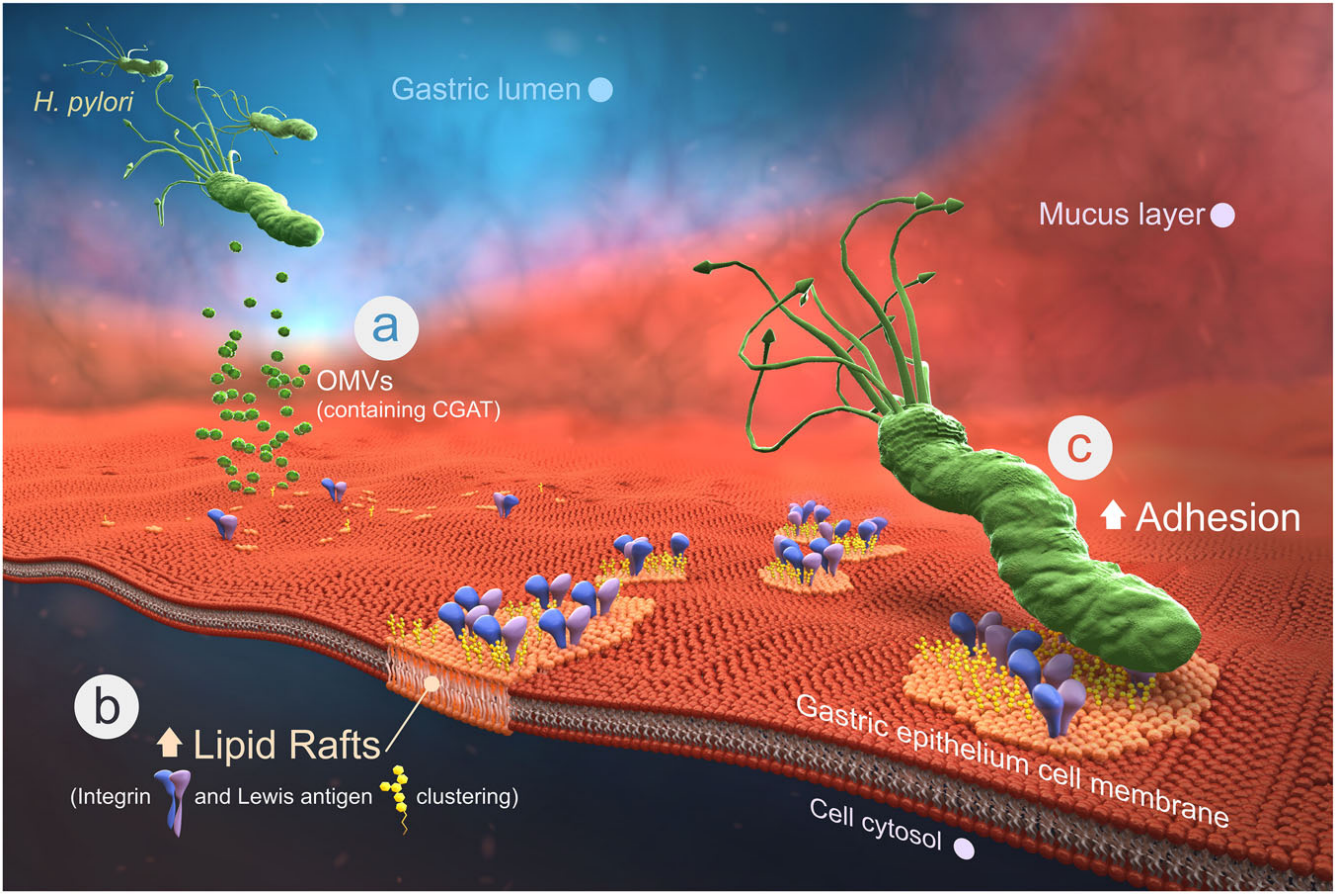
Schematic presentation demonstrates how CGAT is involved in *H. pylori* pathogenesis. **a** *H. pylori* secretes OMVs to deliver the enzymes CGAT and CGT to the host epithelial cells. **b** Upon translocation, the two enzymes produce human lipid-containing CAGs to enhance lipid raft clustering. **c** The integrins and Lewis antigens then gather at the raft region, leading to great enhancement in the bacterial adhesion. Therefore, CGAT inhibitor is able to blockade the adhesion, which sheds light on the discovery of a potentially new target to eradicate *H. pylori* infection.

The amiodarone-based anti-adhesion seems to offer an alternative for antimicrobial therapy. CGAT, the product of *hp0499*, was originally proposed to be an outer membrane phospholipase A that shares 19.7% sequence identity and 35.4% similarity with *E. coli* outer membrane phospholipase A. Our result indicated that CGAT is indeed a phospholipase A1, but the enzyme additionally catalyzes the acyltransfer to the 6′-OH of CG, resulting in the formation of CAG. In contrast to most bacterial infections where bacterial molecules are propagated to the host cell via passive diffusion or direct cell contact, our result represents a very distinctive scenario. The two enzymes CGT and CGAT are packaged in the OMVs and shipped to the host cell (Fig. 6a). Upon fusion of OMVs with the host cell membranes, the two enzymes are released to directly utilize the necessary substrates to form CAGs in the host cell. Moreover, CAG can be considered as a modified version of cholesterol to modulate the features of the host membranes, including lipid rafts formation and clustering. This helps to gather several adhesion molecules around the rafts region, such as Lewis antigens and integrins α5 and β1 (Fig. 6b). The resulting enhanced interactions thus help *H. pylori* to adhere much better (Fig. 6c, Supplementary Movie 2). As a consequence, our previous^14^ and current studies thus demonstrate the intriguing interplay between *H. pylori* and gastric epithelial cell. The bacteria not only hijack cholesterol and PE from the host to make CAG but also leverage the OMVs to deliver the biosynthetic machinery for the direct and efficient synthesis in the host cell. This leads to a dramatic change on the host cell surface; the enhanced lipid rafts clustering further promotes a higher level of bacterial adhesion.

## Discussion

In this study, we identified CGAT as a steryl glycoside acyltransferase. Although this enzyme has been reported to exist in a number of organisms, there is no gene information available^20,21^. The cloning, expression and characterization of CGAT, to the best of our knowledge not only stands for the first in-depth study of a steryl glycoside acyltransferase but also provides useful information to identify enzymes of the same category and understand their functional roles in other organisms.

In the *H. pylori* pathogenesis, the proteins of *cag*-pathogenicity island are associated with integrins α5 and β1 of epithelial cells^11,22^, which induces T4SS assembly and the subsequent injection of CagA. Given that integrins are generally resided in the basolateral side of epithelial cells, it is intriguing to know the reason why the integrins can interact with the T4SS of *H. pylori*. Hecht and co-workers^23^ found that during the infection of *E. coli*, integrin β1 translocated to the apical side of intestinal epithelial cells after the destruction of tight junction proteins. *H. pylori* was shown to impair the tight junction^24^. The bacteria secreted the serine protease HtrA to open cell-to-cell junctions through cleavage of junctional proteins, such as occludin, claudin-8, and E-cadherin in polarized epithelial cells. The opening allowed *H. pylori* to move into the intercellular space between adjacent cells and eventually reach to basolateral locations^25^. As a consequence, it is intriguing to examine if and how the presence of CAG correlates with the impairment of tight junction and junctional proteins.

High cholesterol level is known in association with cardiovascular diseases (CVDs), such as atherosclerosis^26–30^. Therefore, it is intriguing to understand if the cholesterol uptake by *H. pylori* correlates with CVDs; especially the link of *H. pylori* seropositivity to CVD has been described in several reports^31–35^. How the infection of *H. pylori* influences CVD, however, remains unclear. CAG, considered to be a modified version of cholesterol, was shown to promote lipid rafts clustering in gastric epithelia in this and previous studies^14^. Caveolae, representing cholesterol- and sphingolipid-enriched invaginations of the plasma membranes, were identified as a potential regulator of vascular dysfunction and heart disease^36–39^. Moreover, our observation indicated that CAG is rather stable under cell culture conditions, implicating that CAG may not be only available in the infected gastric epithelia, and that their dissemination could promote caveolin-related lipid rafts clustering and thus lead to vascular dysfunction and coronary heart disease. Nevertheless, the levels of CAGs are dynamic depending on the surrounding nutrient condition, not mentioning that different *H. pylori* strains were found to produce various levels of CAGs. This might explain why there are contradictory observations reporting little or no relevance between *H. pylori* infection and CVD^40–42^.

Adherence of *H. pylori* to the gastric epithelium rendered *H. pylori* 100–1000 times more resistant to antibiotics than non-adherent bacteria^43^. The enhanced adhesion of *H. pylori* was observed in epithelial degeneration and in the presence of mucin depletion symptoms when the patients suffered from gastritis^44^. Several reports demonstrated that the prevention of bacterial adhesion was able to reduce bacterial survival and virulence^45,46^; especially the number of bacteria and the related symptoms were significantly diminished^47–49^. Moreover, a recent study showed that cholesterol-derived glucosides were related to antibiotic resistance^50^, which was further supported by the extremely high levels of CAGs in the MDR strains that were clinically isolated (Fig. 5i). Since anti-adhesion impedes the binding of bacteria without affecting microbial viability, little or no pressure is applied to select between the wild-type bacteria and mutants. An anti-adhesion therapy thus possibly minimizes the occurrence of drug resistance. It is extremely difficult, however, to develop multiple inhibitors to prevent the bindings of all adhesion molecules given that bacteria usually have several ways to adhere to the host cells. In this study, the CAG-induced clustering of lipid rafts appears to be the driving force to intensify the bacteria–host cell interactions. The development of CGAT inhibitors turns out to be superior to those directly blocking any of the aforementioned interactions. Intriguingly our result indicated that the bacterial adhesion became very limited (Fig. 5d, k) once the CGAT activity was inhibited. Taken together, there are several advantages associated with CGAT inhibitors, such as inhibition of bacterial adhesion, reduction of the OMV fusion to the host cell, and less likely to develop drug resistance. Our discovery of amiodarone thus becomes valuable because this molecule or other CGAT inhibitors can be utilized in conjunction with antibiotics, especially when facing the emerging threat of antibiotic resistance.

## Methods

### *H*. pylori strains and bacterial cultures

*H. pylori* was grown on CDC Anaerobic Blood Agar plates (Becton Dickinson) under microaerobic (Anaeropack Campylo System, Mitsubishi Gas Chemical, Tokyo, Japan) conditions at 37 °C for 2 days. For liquid cultures, *H. pylori* was grown in Brucella broth (Difco) containing 0.2% β-cyclodextrin (Sigma), 10% fetal bovine serum, and 1% IsoVitaleX (Becton Dickinson) for 60 h. For kanamycin-resistant strain selection, 20 μg ml^−1^ kanamycin was used. For chloramphenicol-resistant strain selection, 5 μg ml^−1^ chloramphenicol was used. The knockout strains were generated from *H. pylori* 26695 by inserting antibiotic-resistance cassettes into the target genes via homologous recombination. For knock out of *hp0421* (ΔCGT), chloramphenicol-resistance cassette was used. For knock out of *hp0499* (ΔCGAT), *hp0420* and *hp0935*, the kanamycin-resistance cassette was used. Three genes (*hp0420*, *hp0935,* and *hp0499*) were selected as the proposed candidates to encode CGAT. *hp0420* was related to acyl carrier protein (ACP)-dehydratase/CoA or thioesterase, while *hp0935* was considered to be an *N*-acetyltransferase in *H. pylori*. *hp0499* was previously reported as outer membrane phospholipase A1 in *H. pylori*. Our data shown in Supplementary Fig. 1 indicated *hp0499* to be CGAT. To complement the CGAT mutation, a CGAT-knock-in strain was generated from the strain of ΔCGAT by inserting one intact *hp0499* gene with a chloramphenicol-resistance cassette to the upstream of the mutant *hp0499* gene locus described above. The correct transformants were obtained by selection with chloramphenicol and kanamycin and then verified by PCR. Clinical isolated multiple drug-resistant (MDR) strains, including 4923, 4974, 5004, 4925, 5033, 4973, 5024, 5022, 4962, and 4955 were obtained from National Taiwan University Hospital, Taiwan. All these MDR strains are resistant to clarithromycin, metronidazole, and levofloxacin. The resistance breakpoints for clarithromycin, metronidazole and levofloxacin were defined as >0.5, >8, and >1 μg ml^−1^, respectively^51^.

### Cell lines and cell culture

Mammalian cells were maintained within a humidified environment and under 5% CO_2_ at 37 °C. AGS (human gastric epithelial, female) and KATO III (human neoplastic epithelial, male) cells were maintained in Dulbecco’s modification of Eagle’s medium (Gibco, Invitrogen) supplemented with fetal bovine serum (10%; HyClone) and penicillin/streptomycin (1%; Biological Industries). Both AGS and KATO III cell lines were obtained from the American Type Culture Collection (ATCC) and were certified by ATCC. ATCC uses morphology-, karyotyping-, and PCR-based approaches to confirm the identity of both cell lines. Cell lines were tested negative for mycoplasma using PCR-based approach. They are not listed in the database of commonly misidentified cell lines.

### Antibodies

The antibodies used in this paper are shown as follows. Anti-*H. pylori* antibody (Abcam, ab20459) was validated and published by Rossez et al.^52^. Rat anti-integrin β1 clone AIIB2 (Merck, MABT409) was validated its functionally blocking antibody by abrogating AGS cell attachment to fibronectin^53^. This antibody was validated its immunocytochemistry activity in AGS cells^53^. Rat anti-integrin α5 clone BIIG2 (DSHB, AB 528155) was validated its blocking function by Damsky and co-workers^54^. This antibody was validated its immunocytochemistry activity by Fisher and co-workers^55^. Mouse anti-blood group Lewis^b^ antibody (IgM) T218 (Santa cruz, Sc-59470) was validated and published by Taniguchi et al.^56^. Mouse anti-CD15s (sialyl Lewis^x^) that is a monoclonal antibody unconjugated clone CSLEX1 (BD Biosciences, 551344) was validated and published by Walz et al.^57^. Anti-*H. pylori* Cag antigen IgG fraction (monoclonal) (Austral Biologicals, HPM-5001-5) and anti-*H. pylori* Cag antigen IgG fraction (polyclonal) (Austral Biologicals, HPP-5003-9) were validated and published by Tegtmeyer et al.^25^. Mouse anti-phosphotyrosine antibody, clone 4G10 (Sigma Millipore, 05-321) was validated and published by Shuai et al.^58^. Polyclonal rabbit anti-*H. pylori* CGAT-specific antibody was created in this study and validated by comparing with the positive and negative controls.

In addition, the secondary antibodies are shown as follows: Goat anti-rabbit IgG (pre-adsorbed to 10 nm gold) (Abcam, ab27234), goat anti-rabbit IgG H&L (HRP) (Abcam Ab6721), anti-GAPDH antibody for mAbcam 9484 loading control (HRP) (Abcam Ab9482), goat anti-mouse IgG H&L cross-adsorbed secondary antibody (Alexa Fluor 488) (Thermo Fisher, A-11001), goat anti-mouse IgG (HRP) (Santa Cruz, sc-2005), goat anti-rabbit (Alexa Fluor 488) (Invitrogen A11034), goat anti-rat IgG H&L cross-adsorbed secondary antibody (Alexa Fluor 488) (Thermo Fisher, A-11006), and goat anti-mouse IgM CFL488 (Santa cruz, sc-395784).

### Sample preparation of *H. pylori* cholesteryl glucosides

First, 50 μM azido-cholesterol was added to the culture of *H. pylori*. Then, Folch partitioning was performed using a chloroform/methanol (1/2) extraction for 1 h and another round of chloroform/methanol (2/1) extraction for 1 h, followed by an extraction with 0.9% KCl. The organic layer was evaporated to give a dried residue that was then subjected to a click reaction with 0.25 mM alkyne-dye (4-*N*-methylamino-1,8-napthalimidopropyne, abbreviated as MAN) in the presence of 1.25 mM tris(benzyltriazolylmethyl)amine (TBTA), 12.5 mM sodium ascorbate, and 0.25 mM CuSO_4_ at room temperature for 1 h. After the samples were dried, they were stored at −20 °C until HPLC or MS analysis.

### Analysis of cholesteryl glucosides

These experiments were performed as previously described^14^. For ultrahigh-performance liquid chromatography (UPLC, Waters)-MS (Orbitrap Elite, Thermo), a CSM C-18 column was used with a gradient from 30% acetonitrile (ACN) with 20 mM ammonium acetate to a mixture of 90% isopropyl alcohol and 10% ACN with 20 mM ammonium acetate. The mass spectrometer was operated in positive electrospray ion mode set to one full FT-MS scan (*m*/*z* 200–1600; 15,000 resolution) and switched to different FT-MS product ion scans (15,000 resolution) for the different precursors of cholesteryl glucoside derivatives. Both collision-induced dissociation and higher energy collisional dissociation were utilized to perform the fragmentation. For sensitive quantitation, multiple reaction monitoring was performed, which detected the fragment of MAN at 366.16 *m*/*z*.

### Protein overexpression and purification

Plasmids were transformed into *E. coli* BL21 (for CGT and CGAT) for protein overexpression. Isopropyl β-d-1-thiogalactopyranoside at a concentration of 0.4 mM was used to induce overexpression at 16 °C for 16–24 h. *E. coli* cells were harvested by centrifugation at 6000 r.p.m. with a JLA-8.1000 rotor (Beckman). For lysis, pellets were suspended in 20 mM Tris buffer with 200 mM NaCl and 1 mM EDTA, pH 8.0. The cells were then lysed by a cell disruptor (Constant Systems) for 5–10 times. The lysates were centrifuged at 1500 *g* for 10 min to remove cell debris. Ultracentrifugation was then applied at 40,000 r.p.m. with a Type 45 Ti Rotor (Beckman) for 1 h to obtain the membrane fraction. The pellets were stirred overnight in 20 mM Tris buffer with 200 mM NaCl and 1% n-dodecyl-β-d-maltopyranoside at pH 8.0 and 4 °C. The resulting mixture was applied for ultracentrifugation again to obtain the soluble membrane proteins in the supernatant. Since all the constructs contained His-tags, a Ni-NTA column was used to purify the target proteins. Buffers containing 0.04% DDM and 900 mM imidazole were used to elute the target proteins. Fractions from purification were analyzed by SDS-PAGE. The proteins were further concentrated by 10 kD Amicon Ultra-15 Centrifugal Filter Units (Millipore) and quantitated by a DC™ protein assay kit (Bio-Rad). All the proteins were stored at −80 °C in the presence of 20% glycerol.

### Cell fractionation of *H. pylori*

These experiments were performed as previously described^15,59–61^ with some modifications. Briefly, *H. pylori* was growth for 2 days and was washed with phosphate-buffered saline (PBS) for three times and re-suspended in 50 mM HEPES and 0.2 M NaCl buffer. To lyse bacteria, sonication was performed for 30 min alternatively repeated with sonication (10 s) and rest (10 s). The lysed cells were centrifuged at 2000 *g* for 20 min to remove unlysed cells and other large cell debris. The resulting supernatant was subjected to ultracentrifugation for 90 min at 82,000 *g* to obtain the cytoplasmic fraction (supernatant). Pellet containing membrane fractions was further separated to give inner and outer membrane fractions by differential solubilization with *N*-lauroylsarcosine (Sigma). Resuspension of this pellet by 500 µl of 50 mM Tris-HCl (pH 7.5) with 0.6% (w/v) *N*-lauroylsarcosine was performed. After 20 min of *N*-lauroylsarcosine incubation and 82,000 *g* ultracentrifugation for 90 min at 4 °C, the inner membrane fraction was obtained in the supernatant. The pellet containing outer membranes was re-suspended in 500 µl of 50 mM Tris-HCl (pH 7.5) buffer and stored for further experiment.

### OMV purification

These experiments were performed as previously described^62^. *H. pylori* liquid cultures were harvested by centrifugation at 6000 *g* for 30 min. The medium was filtered by 0.45 μm filters and then subjected to ultracentrifugation at 36,000 r.p.m. in a Type 45 Ti Rotor for 1 h. Pellets were washed with PBS and combined. After another step of ultracentrifugation, the pellets were re-suspended in F12 medium, and protein was quantitated by a DC™ protein assay kit. Samples were stored at −20 °C until used. For imaging of OMVs, OMVs were stained with Vancomycin BODIPY™ FL Conjugate (Thermo Fisher, v34850).

### Activity assays of CGAT

CGAT activity was measured by MS-based determination of CAG and lysophosphatidylethanolamine. First, 10 μl of 1,2-dimyristoyl-*sn*-glycero-3-phosphoethanolamine (PE) (5 mM in ethanol) and 2 μl of CG-MAN (2 mM in ethanol) were evaporated. Second, buffer at a final volume of 100 μl containing 100 mM formic acid and 10 mM EDTA was mixed with dried PE and CG. Third, recombinant CGAT was added to the reaction buffer to a final concentration of 100 nM, followed by sonication of the resulting mixture for 5 s. The reaction was incubated for 16 h before the addition of CaCl_2_. The resulting mixture was further incubated for 2 h. The reaction was terminated and simply extracted by adding 10 μl of EDTA (0.5 M) and 90 μl of KCl (0.9%) with chloroform/methanol (1/2). The aqueous layer was then extracted again with chloroform/methanol (2/1), and the organic layers were combined and evaporated to give dried residues that were stored at −20 °C until MS analysis.

### Protein identification

Protein samples were analyzed by SDS-PAGE. After normal staining and destaining procedures, the desirable bands were cut into small slices and completely destained with 50% ACN in 25 mM ammonium bicarbonate (ABC) buffer. Reduction was performed by using 50 mM dithiothreitol in 25 mM ABC buffer at 37 °C for 1 h. The alkylation was performed by using 100 mM iodoacetamide in 25 mM ABC buffer at room temperature for 1 h in dark. Gel slices were then washed four times with 50% ACN in 25 mM ABC buffer for 15 min each and dried after the addition of 100% ACN. Lys-C and Trypsin in 25 mM ABC buffer were added sequentially to digest the proteins for 16 h at 37 °C. Peptides were extracted by sonication of the gel slices in 50% ACN and 5% trifluoroacetic acid for 2 min (repeatedly sonicated 10 s and relaxed 10 s) twice. After centrifugation, the supernatants were transferred into new tubes and evaporated. A Zip-Tip desalting procedure was then performed. After the samples had been washed with 0.1% formic acid, they were eluted with 50% ACN and 0.1% formic acid. The samples were then dried and subjected to proteomic analysis. Mascot database searching was used for protein identification. The data were selected with a false discovery rate (FDR) <1%. The searching was performed on the concatenated MGF files with an ion score cutoff of 50 and a significance threshold of *p* < 0.05. Only peptides with ion scores over 50 were considered (bold peptide), and only proteins with at least one unique peptide (red bold in Mascot) were considered. The mass spectrometry proteomics data have been deposited to the ProteomeXchange Consortium via the PRIDE^63^ partner repository with the dataset identifier PXD017518 and 10.6019/PXD017518.

### Cryo-TEM imaging

Holey carbon film-covered 300 mesh copper grids (HC300-Cu, PELCO) were glow-discharged in an (Ar, O_2_)-atmosphere for 10 s on the carbon side. Samples (1–10 mg ml^−1^) of 4 μl volumes were pipetted onto grids. Grids were blotted in 100% humidity at 4 °C for 3 s and plunge-frozen into liquid ethane cooled by liquid nitrogen using a Vitrobot (FEI, Hillsboro, OR). Liquid nitrogen was used to store grids. Grids were then transferred to the electron microscope using a cryostage. Images of OMVs within the holes in the carbon film were obtained by using a Tecnai F20 electron microscope (FEI) at 200 keV with a 70 μm objective aperture. For each exposure, the low dose condition was ~20*e*^−^ Å^−2^. Images were taken at 5k or 50k magnification and 2–3 μm defocus and recorded on a 4k × 4k CCD camera (Gatan, USA).

### Cell preparation procedure

AGS cells (5 × 10^5^) or KATO III cells (10^6^) were seeded onto 35 mm dishes in Dulbecco’s modified Eagle's medium (Gibco, Invitrogen) at 37 °C for 16 h. Cells were first washed with PBS three times before being treated with 5 mM methyl-β-cyclodextrin in serum-free Ham’s F-12 nutrient medium (F12 meidum) for 1 h. After washes with PBS, cells were collected for further experiments.

### Immunofluorescence microscopy

For lipid raft staining, AGS cells were subjected to the cell preparation procedure and incubated with compounds for 1 h; enzymes for 8 h; OMVs from different *H. pylori* strains for 1, 2, 6, or 8 h; or different amounts of OMVs for 6 h. Lipid rafts (GM1) in AGS cells were then labeled with Alexa Fluor 594-conjugated cholera toxin subunit β. Fluorescence microscopy was performed to visualize the clustering of GM1. For CGAT staining, AGS cells were subjected to the cell preparation procedure and treated with OMVs or co-cultured with *H. pylori* at a multiplicity of infection (MOI) of 50 for 8 h. Cells were then fixed (2% formaldehyde) and stained with a specific antibody for CGAT (1:500). Immunofluorescence microscopy was performed to visualize CGAT (Alexa Fluor 488, green). Nuclei were counterstained with DAPI (blue). For integrin or Lewis antigen staining, AGS or KATO III cells were subjected to the cell preparation procedure and treated with CAG(18:0) for 1 h or OMVs for 8 h. Lipid rafts (GM1) in cells were then labeled with Alexa Fluor 594-conjugated cholera toxin subunit β for 10 min. Cells were then fixed (2% formaldehyde) and stained with a specific antibody for integrin α5 (BIIG2; 1:125) or β1 (AIIB2; 1:400) or Lewis^b^ antibody (T218; 1:100) or sialyl Lewis^x^ antibody (CSLEX1; 1:1000) at 4 °C for 16 h. Immunofluorescence microscopy was performed to visualize integrins and Lewis antigens (Alexa Fluor 488, green).

### Live-cell imaging of AGS cells

AGS cells were subjected to the cell preparation procedure and incubated with Alexa Fluor 594-conjugated cholera toxin subunit β (Molecular Probes, USA) for 10 min, washed with PBS three times, and then treated with 200 μg of OMVs that had been labeled by Bodipy FL-vancomycin (1 μM; Green fluorescence) at 37 °C for 8 h. The images of serial optical sections (1024 × 1024 pixels) were obtained under a ×100 Plan-NEOFLUAR lens (laser 543 nm) at 5 min intervals using a Zeiss LSM 510 confocal microscope. The shown live-cell images were representative of three independent experiments.

### Immunoprecipitation and blotting

These experiments were performed as previously described^14^. AGS cells (6 × 10^5^) were seeded in a six-well tissue culture dish in Dulbecco’s modified Eagle's medium (Gibco, Invitrogen) and incubated at 37 °C for 16 h. These cells were then treated with 5 mM methyl-β-cyclodextrin in serum-free medium for 1 h and subsequently treated with compounds for 1 h, enzymes for 8 h, or OMVs from different *H. pylori* strains for 8 h. These cells were infected with *H. pylori* 26695 at an MOI of 50 at 37 °C for 1 h. CagA translocation and CagA tyrosine phosphorylation were detected by immunoblotting. After infection of AGS cells with *H. pylori*, cells were washed three times with PBS and collected with a cell scraper. To detect the CagA internalized into AGS cells, saponin was used to lyse AGS cells while keeping the bacteria intact. The AGS cells were gently lysed with PBS containing 0.1% saponin, protease inhibitor cocktail (Calbiochem, USA), and phosphatase inhibitor cocktail (Calbiochem, USA) for 10 min at room temperature. The cell debris and intact bacteria were separated from the soluble fraction by centrifugation (6000 *g* for 5 min), and the solute was filtered with a 0.22-μm-pore filter to yield human cell proteins and the internalized CagA. The saponin lysis procedure was also performed on *H. pylori* 26695 alone to make sure that this procedure did not release cytoplasmic proteins from *H. pylori*. Filtrate (100 μg) was immunoprecipitated by using 10 μg of rabbit anti-CagA polyclonal antibody (Austral Biologicals, USA) and 30 μl of Protein A Plus Agarose (Thermo Scientific, USA) at 4 °C overnight. The obtained precipitates were washed three times with immunoprecipitation buffer (25 mM Tris, 150 mM NaCl, 5 mM EDTA, and 1% TX-100, pH 7.4) and once with TBS buffer (50 mM Tris, 150 mM NaCl, and 1 mM CaCl_2_, pH 7.4) and boiled in SDS-PAGE sample buffer (62.5 mM Tris, 2% SDS, 10% (v/v) glycerol, and 0.05% brilliant blue R, pH 6.8) at 95 °C for 10 min. The resulting samples were resolved by 6% SDS-PAGE, and the bands were transferred onto polyvinylidene difluoride membranes (Millipore, USA). The membranes were blocked with 5% (w/v) BSA in TBS buffer containing 0.01% Tw-20 at room temperature for 1 h and incubated overnight with mouse monoclonal anti-CagA (Austral Biologicals; 1:2000), mouse monoclonal anti-phosphotyrosine (4G10, Millipore; 1:2000) or mouse monoclonal anti-GAPDH (ab9482, Abcam, UK; 1:5000) at 4 °C. The blots were washed and incubated with HRP-conjugated secondary antibodies (Santa Cruz Biotechnology, USA) at a 1:5000 dilution, and the proteins of interest were visualized using an enhanced chemiluminescence assay (WBKLS0500, Millipore).

### Flow cytometry analysis

Cells treated with compounds, enzymes, OMVs, blocking antibodies, or inhibitors were infected with *H. pylori* 26695 (MOI = 50) at 37 °C for 1 h. After the cells were washed to remove non-adherent bacteria, they were detached with 2 mM EDTA and fixed with 2% formaldehyde. Cells with plasma membrane-associated *H. pylori* bacteria were stained with rabbit anti-*H. pylori* antibody (Abcam, 1:1,000) at 4 °C for 16 h and then washed with PBS. The secondary antibody was fluorescein isothiocyanate (FITC)-conjugated goat anti-rabbit antibody (Invitrogen, 1:1000), which was applied at 4 °C for 1 h, and the samples were then washed with PBS. Cells were analyzed by a flow cytometry system (BD FACSCalibur Calibur Flow Cytometer 4 Color). BD FACStation Software (Version 3.3) was used for the data acquisition. Adherence was quantitated in terms of the proportion of cells with adherent *H. pylori*. At least over 10,000 cells were collected. For gating strategy, as shown in Supplementary Fig. 5, cell debris was first removed by gating the main cell population using the FSC/SSC gating. Mock-treated cells (cells only without bacterial infection) that had been treated with the same staining procedure as aforementioned were used to distinguish the boundary between the positive or negative staining populations. An identical threshold was applied for all samples within the same cell line.

### Statistics and reproducibility

The results are expressed as the mean ± SD (standard deviation). For the graphs, data were combined from at least three biological independent experiments. Statistical significance between two samples was tested by an unpaired *t-*test using Prism 8.0 software (GraphPad Software, La Jolla, CA). All statistically significant differences are indicated with asterisks; ***p < 0.001, **p < 0.01, *p < 0.05 (not significant, *p* > 0.05).

### Reporting summary

Further information on research design is available in the Nature Research Reporting Summary linked to this article.

## Supplementary information

Supplementary Information

Description of Additional Supplementary Files

Supplementary Movie 1

Supplementary Movie 2

Supplementary Data 1

Supplementary Data 2

Reporting Summary

## Data Availability

The data that support the findings of this study are available from the corresponding author on request. All source data in the main figures are available in Supplementary Data 1 and 2. The mass spectrometry proteomics data have been deposited to the ProteomeXchange Consortium via the PRIDE^63^ partner repository with the dataset identifier PXD017518 and 10.6019/PXD017518. Full blots are shown in Supplementary Information.
