# Supplementary material for: Cholesteryl α-D-glucoside 6-acyltransferase enhances the adhesion of Helicobacter pylori to gastric epithelium
Source: Commun Biol. 2020 Mar 13;3:120. doi: 10.1038/s42003-020-0855-y (PMC7069968; doi:10.1038/s42003-020-0855-y)
Supplement: Supplementary file 1 — Supplementary Information [file 42003_2020_855_MOESM1_ESM.pdf]

Supplementary Information

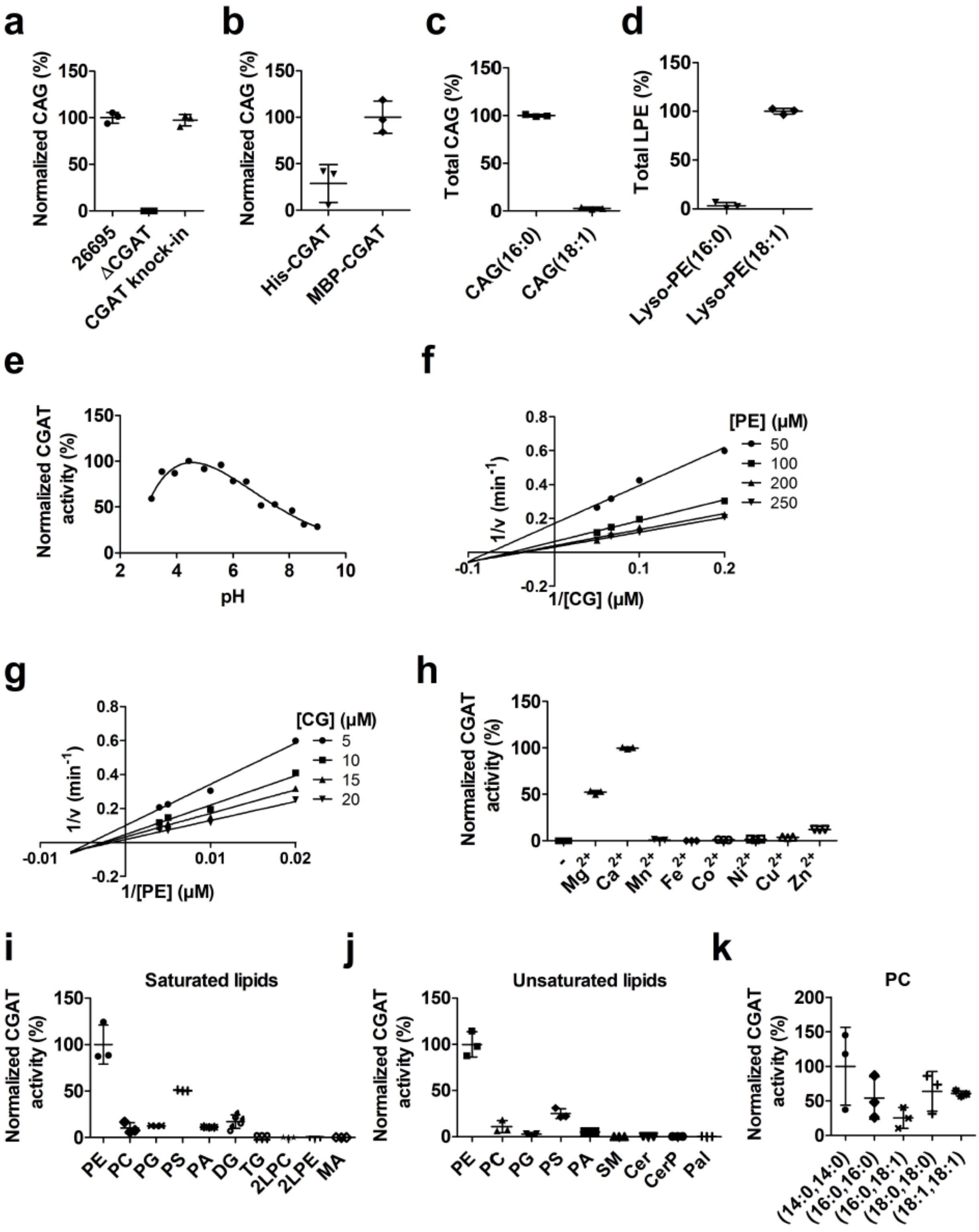

Supplementary Figure 1. Characterization of recombinant CGAT

a, Levels of CAGs in *H. pylori* 26695, a CGAT-deficient strain (ΔCGAT), and a

CGAT-knock-in strain. See Methods section regarding the procedure to prepare the strains of ΔCGAT and CGAT-knock-in. Data were measured by LC-MS analysis.

b, Relative activity of recombinant MBP-CGAT and 6 x His-tagged CGAT. Both recombinant CGATs were overexpressed in *E. coli*. The recombinant CGAT was incubated for 2 h with the mixture of CG and PE(14:0,14:0) at pH 4.5. The resulting CAG products were analyzed by LC-MS analysis.

c, and **d,** The reaction of CGAT transfers a specific acyl chain from PE to CG. The recombinant protein CGAT was incubated for 2 h with the mixture of CG and PE (d16:0/18:1; i.e., the PE contained deuterium-labeled palmitic acid (16: 0) and unlabeled oleic acid (18:1) at a1- and a2-positions, respectively). The resulting products CAG and lysophosphatidylethanolamine (lyso-PE), were analyzed by LC-MS analysis and the relative levels were shown in (**c**) and (**d**), respectively.

(**e**) pH profiles of the enzyme activity in MBP-CGAT. The reactions were performed as (**b**) at different pH. The resulting CAG products were analyzed by LC-MS analysis.

f, and **g,** Double reciprocal plots of the CGAT-catalyzed reactions with titration of CG at four concentrations of PE(14:0,14:0) (**f**) or vice versa (**g**). The

reactions were performed as (**b**). Data were analyzed by LC-MS analysis.

h, Metal ion dependence of CGAT. The reactions were performed as (**b**). The resulting CAG products were analyzed by LC-MS analysis.

i, Substrate specificity of CGAT to examine various saturated lipids. All lipids contained one, two or three myristoyl chains. The reactions were performed as (**b**). Data were analyzed by LC-MS analysis. Abbreviations: PE, phosphatidylethanolamine; PC, phosphatidylcholine; PG, phosphatidylglycerol; PS, phosphatidylserine; PA, phosphatidic acid; DG, diacylglycerol; TG, triacylglycerol; 2LPC, 1-myristoyl-2-hydroxy-sn-glycero-3-phosphocholine; 2LPE, 1-myristoyl-2-hydroxy-sn-glycero-3-phosphoethanolamine; MA: myristic acid.

j, Substrate specificity of CGAT to examine various unsaturated lipids. The reactions were performed as (**b**). Data were analyzed by LC-MS analysis. All phospholipids contained esters of palmitic acid and oleic acid (at a1- and a2-positions, respectively). Sphingomyelin (SM), ceramide (Cer), and ceramide phosphate (CerP) were N-palmitoyl-D-erythro-sphingolipids (d18:1/16:0). Pal represents palmitic acid.

k, Substrate specificity of CGAT to examine phosphatidylcholines (PCs) of different acyl chains. The reactions were performed as (**b**). Data were

analyzed by LC-MS analysis.

In (**i**-**k**) all lipids were present in 0.5 mM. In (**a-e** and **h-k**), all data were obtained from three replicates and shown as mean ± SD and normalized by scaling the highest data as 100%.

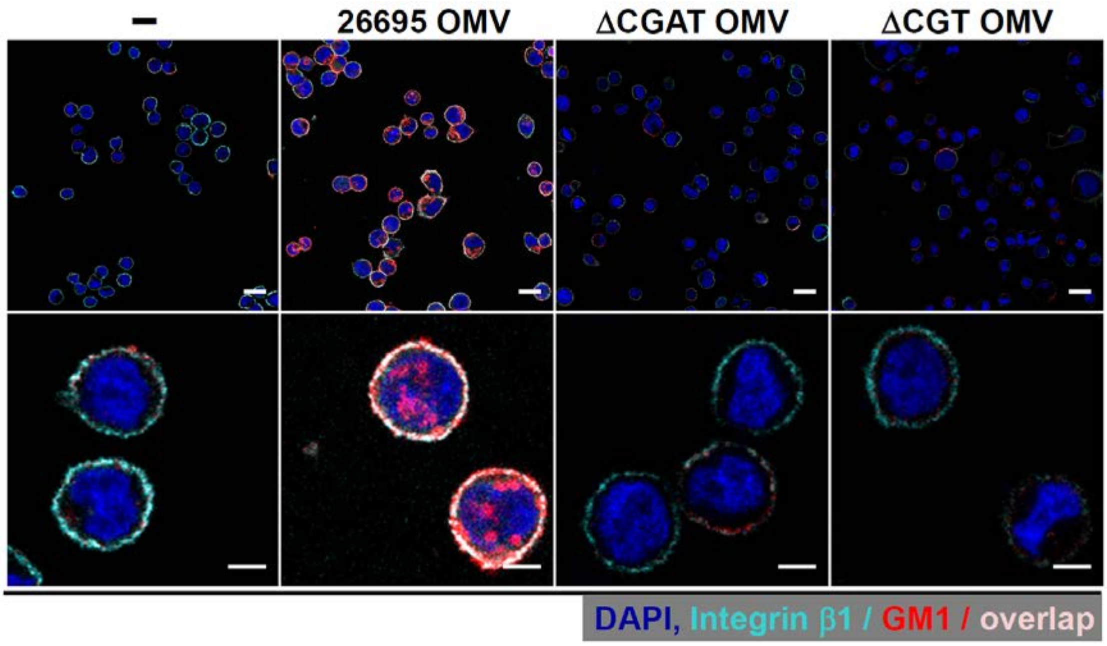

Supplementary Figure 2. Gathering of integrin β1 at the rafts region when KATO III cells were treated with H. pylori 26695 OMVs

KATO III cells were treated with *H. pylori* 26695 OMVs for 8 h, and then cholera toxin B and specific antibodies to label GM1 (red) and integrin β1 (cyan), respectively. Nuclei were counterstained with DAPI (blue). Fair pink color represents the co-localization of lipid rafts and integrin β1. Scale bars denote 20 (upper) and 5 (lower) μm.

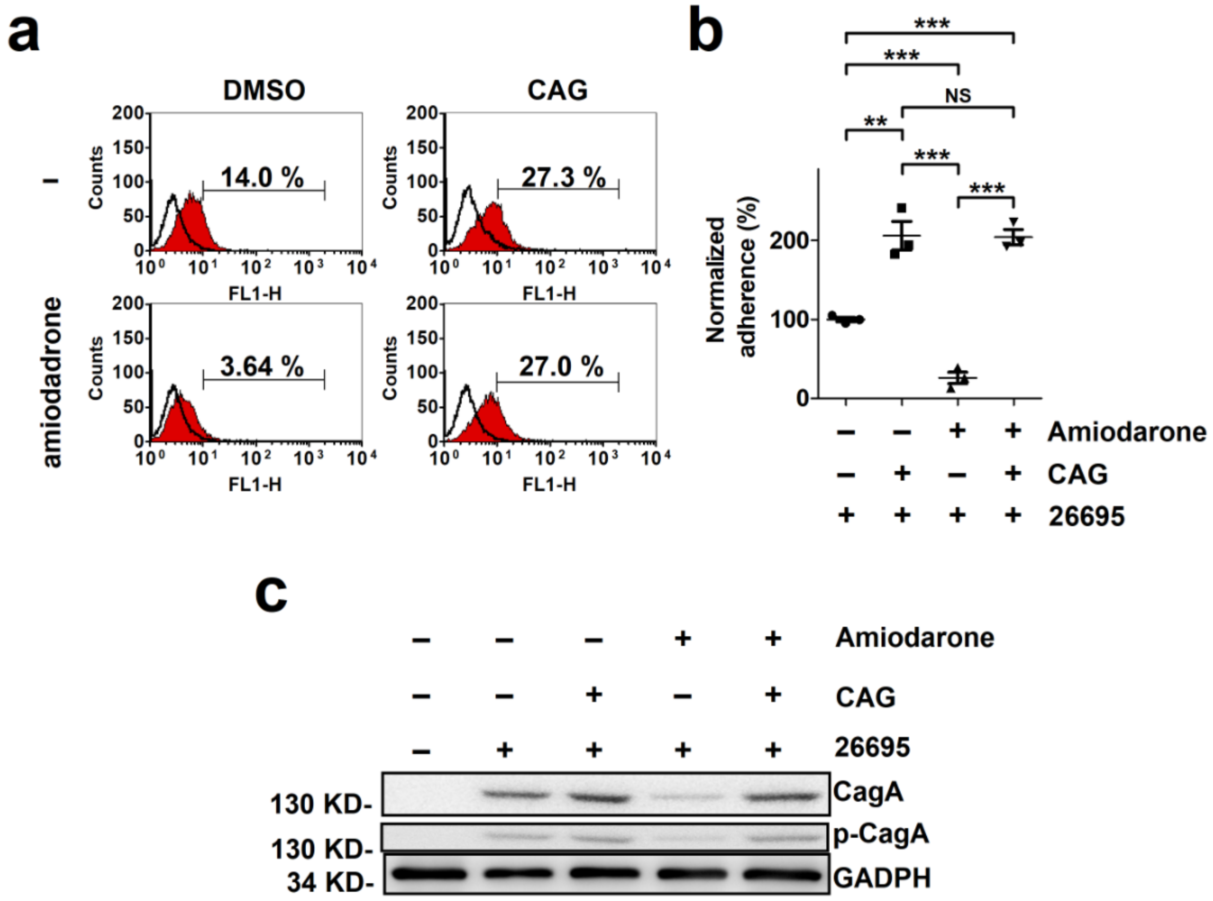

Supplementary Figure 3. Effects of amiodarone on the *H. pylori* adhesion and CagA translocation and the related tyrosine phosphorylation in the presence of CAG(18:0)

(**a-c**) Effects of amiodarone on *H. pylori* adhesion, CagA translocation and CagA tyrosine phosphorylation in the presence of CAG(18:0). After treatment with or without 50 μM amiodadrone in the presence of CAG(18:0) for 2 days, *H. pylori* 26695 was cultured with AGS cells for 1 h. **a,** The degree of adhesion was measured by flow cytometry analysis and quantitated as the proportion of adhered cells with *H. pylori* (%; shown in each plot). **b,** The

quantitation result made in (**a**) was shown as mean ± SD. All statistically significant differences are indicated with asterisks; ***p < 0.001, **p < 0.01 vs. the control group (n = 3). NS represents not significant. **c,** The corresponding CagA translocation and CagA tyrosine phosphorylation were detected by immunoblotting. Uncropped immunoblot images for **c** are provided in Supplementary Figure 4.

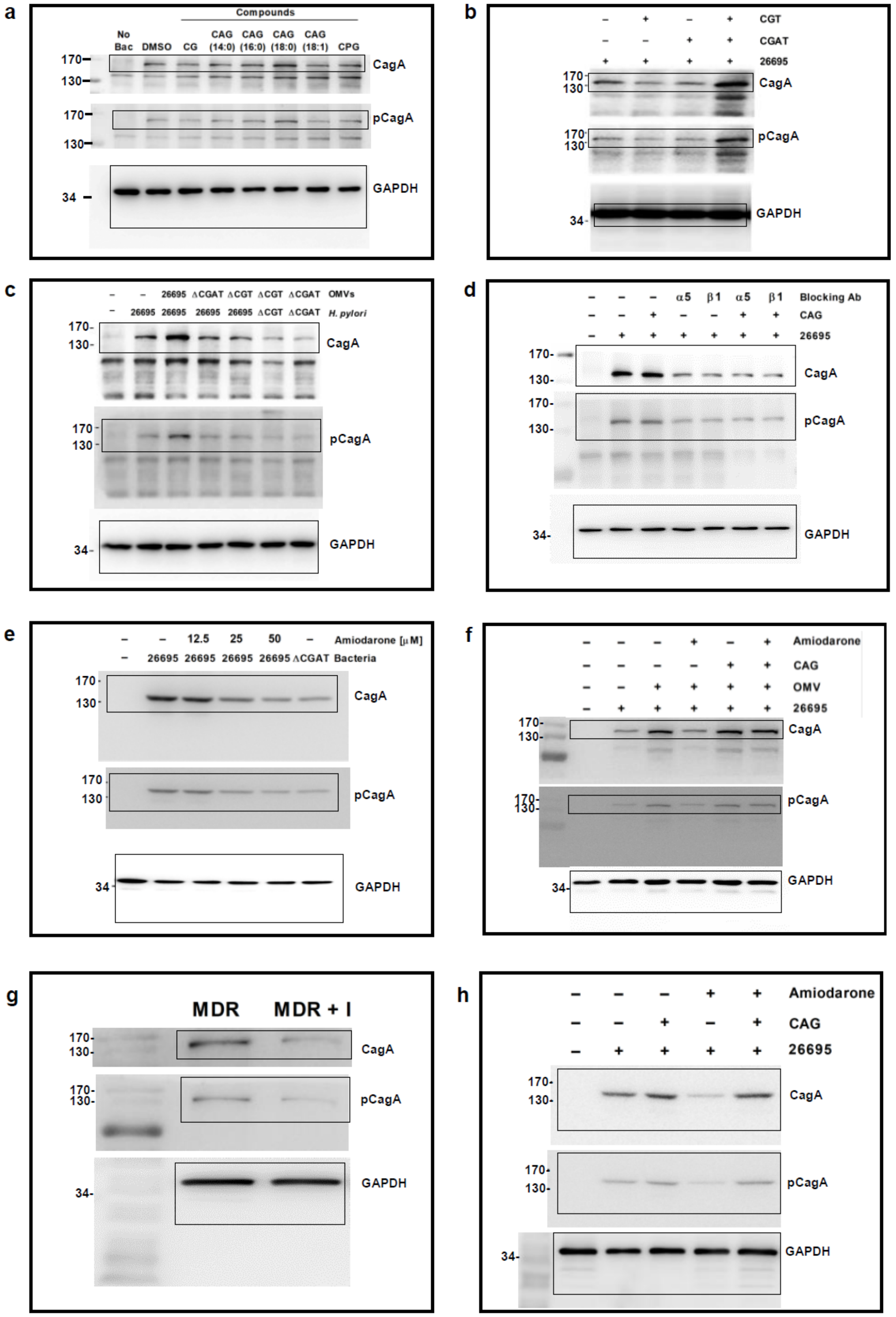

Supplementary figure 4. Full pictures of protein blots presented in the paper. Protein blots are correspond to figure 1e (**a**), figure 3j (**b**), figure 3k (**c**),

figure 4e (**d**), figure 5e (**e**), figure 5h (**f**), figure 5l (**g**) and Supplementary 3c (**h**).

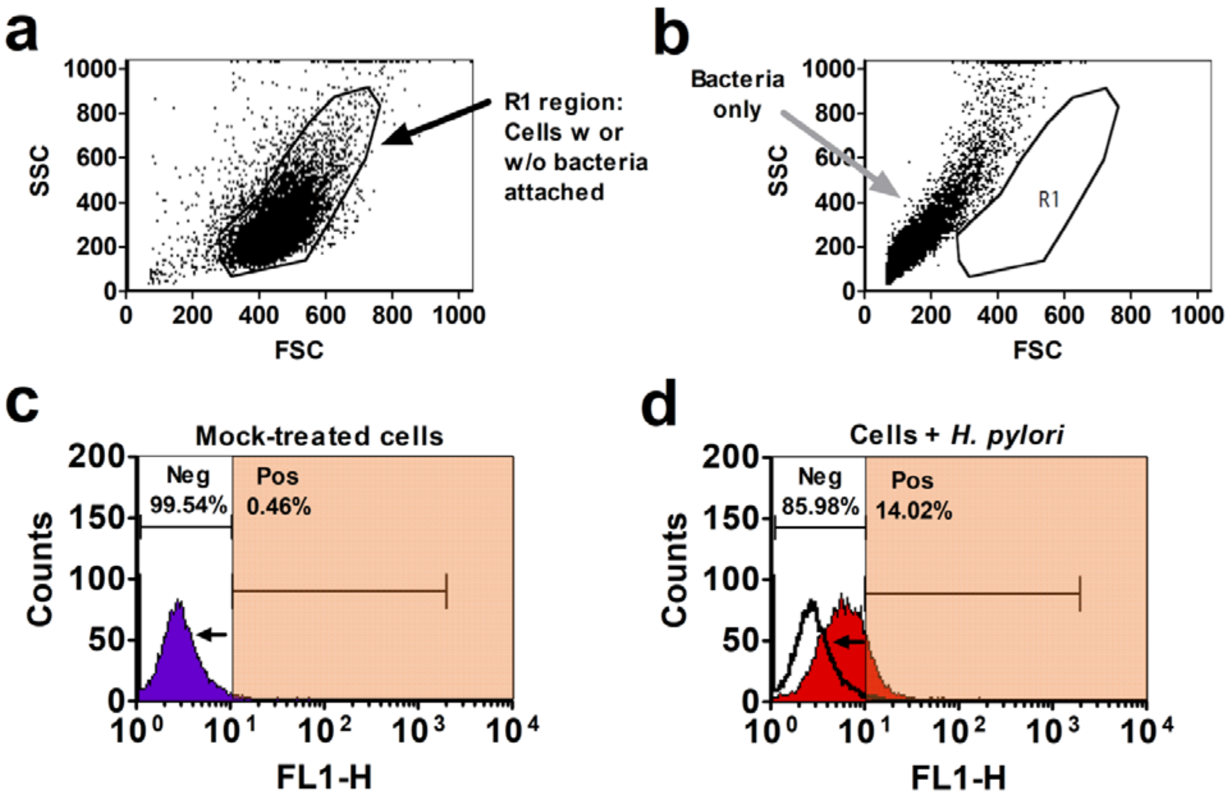

Supplementary Figure 5 shows the gating strategy to identify cells with or without bacterial attachment. In FSC/SSC diagrams (**a**, cells infected by bacteria) and (**b**, bacteria cultured alone), the cells in R1 region were defined, analyzed the fluorescent intensity in (**a**), and to generate the histograms (**c**) and (**d**). (**b**) was used as the negative control. Using this gating strategy, cell debris and bacteria without cell attachment were removed by gating the main cell population. To detect and analyze the cells attached by bacteria (*H. pylori*), a representative histogram for mock-treated cells was shown in (**c**). Mock-treated cells (cells only without bacterial infection) were used to distinguish the boundary between the positive- or negative-staining populations. (**d**) A representative histogram for the cells infected by *H. pylori*

(red region). The population of the cells with bacterial attachment was measured to be 14.02%. Mock-treated cells were also visualized in a transparent peak (indicated by an arrow) that is the same as the peak shown in (**c**). An identical threshold was applied for all samples within the same cell line.

Strains	Total proteins identified	Minimum score	Score of CGT	Score of CGAT
**26695**	416	18	N.D.	39
**ΔCGT**	337	18	N.D.	101
**ΔCGAT**	313	19	N.D.	N.D.

Supplementary Table 1. Numbers of proteins identified from the OMVs of three different *H. pylori* strains. After extraction from the OMVs of *H. pylori* 26695, ΔCGT, or ΔCGAT strains, the protein samples were performed the proteomic analysis. The corresponding protein identification was performed by LC-MS/MS using a linear ion trap-Orbitrap mass spectrometer coupled online with a UPLC system. Mascot was applied for the database search. All the data presented were filtered by false discovery rate (FDR) <1 %. N.D.: Not detected. Detailed mass based proteomics data were available in Supplementary Data 2.
