# Supplementary material for: Cholesteryl α-D-glucoside 6-acyltransferase enhances the adhesion of Helicobacter pylori to gastric epithelium
Source: Commun Biol. 2020 Mar 13;3:120. doi: 10.1038/s42003-020-0855-y (PMC7069968; doi:10.1038/s42003-020-0855-y)
Supplement: Supplementary file 2 — Description of Additional Supplementary Files [file 42003_2020_855_MOESM2_ESM.pdf]

Supplementary Movie 1. Real time imaging of OMV-induced lipid raft clustering in AGS cells

Supplementary Movie 2. A schematic animation shows how CGAT is involved in *H. pylori* pathogenesis

Supplementary Data 1. Source data

All source data in the main figures are available in Supplementary Data 1.

Supplementary Data 2. Mass-based proteomics data
