# Supplementary material for: Cholesteryl α-D-glucoside 6-acyltransferase enhances the adhesion of Helicobacter pylori to gastric epithelium
Source: Commun Biol. 2020 Mar 13;3:120. doi: 10.1038/s42003-020-0855-y (PMC7069968; doi:10.1038/s42003-020-0855-y)
Supplement: Supplementary file 7 — Reporting Summary [file 42003_2020_855_MOESM7_ESM.pdf]

nature research

Corresponding author(s): Chun-Hung Lin

Last updated by author(s): Aug 22, 2019

Reporting Summary

Nature Research wishes to improve the reproducibility of the work that we publish. This form provides structure for consistency and transparency in reporting. For further information on Nature Research policies, see Authors & Referees and the Editorial Policy Checklist.

Statistics

For all statistical analyses, confirm that the following items are present in the figure legend, table legend, main text, or Methods section.

n/a	Confirmed	
☐	☒	The exact sample size (*n*) for each experimental group/condition, given as a discrete number and unit of measurement
☐	☒	A statement on whether measurements were taken from distinct samples or whether the same sample was measured repeatedly
☐	☒	The statistical test(s) used AND whether they are one- or two-sided<br>*Only common tests should be described solely by name; describe more complex techniques in the Methods section.*
☐	☒	A description of all covariates tested
☐	☐	A description of any assumptions or corrections, such as tests of normality and adjustment for multiple comparisons
☐	☒	A full description of the statistical parameters including central tendency (e.g. means) or other basic estimates (e.g. regression coefficient) AND variation (e.g. standard deviation) or associated estimates of uncertainty (e.g. confidence intervals)
☐	☒	For null hypothesis testing, the test statistic (e.g. *F*, *t*, *r*) with confidence intervals, effect sizes, degrees of freedom and *P* value noted<br>*Give P values as exact values whenever suitable.*
☒	☐	For Bayesian analysis, information on the choice of priors and Markov chain Monte Carlo settings
☒	☐	For hierarchical and complex designs, identification of the appropriate level for tests and full reporting of outcomes
☒	☐	Estimates of effect sizes (e.g. Cohen's *d*, Pearson's *r*), indicating how they were calculated

Our web collection on statistics for biologists contains articles on many of the points above.

Software and code

Policy information about availability of computer code

Data collection	No software was used.
Data analysis	Prism and Microsoft Excel (2016 Windows) were used for data analysis when appropriate.

For manuscripts utilizing custom algorithms or software that are central to the research but not yet described in published literature, software must be made available to editors/reviewers. We strongly encourage code deposition in a community repository (e.g. GitHub). See the Nature Research guidelines for submitting code & software for further information.

Data

Policy information about availability of data

All manuscripts must include a data availability statement. This statement should provide the following information, where applicable:

- Accession codes, unique identifiers, or web links for publicly available datasets
- A list of figures that have associated raw data
- A description of any restrictions on data availability

The data that support the findings of this study are available from the corresponding author upon reasonable request.

Field-specific reporting

Please select the one below that is the best fit for your research. If you are not sure, read the appropriate sections before making your selection.

☒ Life sciences ☐ Behavioural & social sciences ☐ Ecological, evolutionary & environmental sciences

For a reference copy of the document with all sections, see nature.com/documents/nr-reporting-summary-flat.pdf

Life sciences study design

All studies must disclose on these points even when the disclosure is negative.

Sample size	Sample size was determined by the number of cells with Cn-B-binding (lipid rafts marker). For the ratios of co-localized cells, sample size was roughly 150 cells.
Data exclusions	No data was exluded
Replication	All attempts at replication were successful
Randomization	In each set of experiments data was collected randomly
Blinding	The researchers were not blinded to sample identity because image collection was conducted using cells from randomly selected fields and raw data collected from the randomly selected fields was used for the analysis.

Reporting for specific materials, systems and methods

We require information from authors about some types of materials, experimental systems and methods used in many studies. Here, indicate whether each material, system or method listed is relevant to your study. If you are not sure if a list item applies to your research, read the appropriate section before selecting a response.

Materials & experimental systems

n/a	Involved in the study	
☐	☒	Antibodies
☐	☒	Eukaryotic cell lines
☒	☐	Palaeontology
☒	☐	Animals and other organisms
☒	☐	Human research participants
☒	☐	Clinical data

Methods

n/a	Involved in the study	
☒	☐	ChIP-seq
☐	☒	Flow cytometry
☒	☐	MRI-based neuroimaging

Antibodies

Antibodies used

1. Anti-Helicobacter pylori antibody, Abcam, ab20459
2. Rat Anti-Integrin Beta1_clone AIIB2, Merck, Cat.#MABT409
3. Rat Anti-Integrin alpha-5_clone BIIG2, DSHB, AB 528155
4. Mouse Anti-Blood Group Lewis b Antibody (IgM) (T218), Santa cruz, Sc-59470
5. Mouse Anti-CD15s (Sialyl Lewis x) Monoclonal Antibody_Unconjugated_Clone CSLEX1, BD Biosciences, 551344
6. Anti-Helicobacter pylori Cag antigen IgG fraction (monoclonal) Austral Biologicals HPM-5001-5
7. Anti H. pylori Cag antigen IgG fraction (polyclonal) Austral Biologicals HPP-5003-9
8. Mouse Anti-Phosphotyrosine Antibody, clone 4G10 Sigma Millipore 05-321
9. Polyclonal rabbit Anti-Helicobacter pylori CGAT-specific antibody This study N/A

10. Goat Anti-rabbit IgG (10nm Gold) preadsorbed, Abcam, ab27234
11. Goat Anti-Rabbit IgG H&L (HRP) Abcam Ab6721
12. Anti-GAPDH antibody mAbcam 9484 loading Control (HRP) Abcam Ab9482
13. Goat anti-Mouse IgG (H+L) Cross-Adsorbed Secondary Antibody, Alexa Fluor 488 Thermo Fisher A-11001
14. Goat anti-mouse IgG-HRP Santa Cruz Biotechnology, USA sc-2005
15. Goat anti-rabbit Alexa fluor 488 Invitrogen A11034
16. Goat anti-Rat IgG (H+L) Cross-Adsorbed Secondary Antibody, Alexa Fluor 488 Thermo Fisher A-11006
17. Goat anti mouse IgM CFL488 Santa cruz SC395784

Validation

1. Anti-Helicobacter pylori antibody (Abcam, ab20459) was validated and published by Rossez Y et al, (Glycobiology 22:1193-206 (2012)).

2. Rat Anti-Integrin Beta1_clone AIIB2 (Merck, Cat.#MABT409) was validated its functionally blocking antibody abrogated AGS cell attachment to fibronectin while causing only a slight inhibition of cell attachment to vitronectin (Hutton, M., et. al. (2010). Infection and Immunity. 78(11):4523-4531). This antibody was validated its immunocytochemistry activity in AGS cells (Hutton, M., et. al. (2010). Infection and Immunity. 78(11):4523-4531).

3. Rat Anti-Integrin alpha-5_clone BIIG2 (DSHB, AB 528155) was validated its functionally blocking function by Damsky CH., et al, (The Journal of cell biology 109.2 (1989 Aug): 877-89.) This antibody was validated its immunocytochemistry activity by Fisher SJ., et al. (The Journal of clinical investigation 89.1 (1992 Jan): 210-22.)

4. Mouse Anti-Blood Group Lewis b Antibody (IgM) (T218) (Santa cruz, Sc-59470) was validated and published by Taniguchi, A., et al. (2000, Biochem. Biophys. Res. Commun. 273: 370-376)

5. Mouse Anti-CD15s (Sialyl Lewis x) Monoclonal Antibody_Unconjugated_Clone CSLEX1, (BD Biosciences, 551344) was validated and published by Walz G., et al. (Science. 1990; 250(4984):1132-1135)

6. Anti-Helicobacter pylori Cag antigen IgG fraction (monoclonal) (Austral Biologicals HPM-5001-5) was validated and published by Tegtmeyer N., et al.(Cell Host Microbe. 2017 Oct 11;22(4):552-560.e5.)

7. Anti H. pylori Cag antigen IgG fraction (polyclonal) (Austral Biologicals HPP-5003-9) was validated and published by Tegtmeyer N., et al.(Cell Host Microbe. 2017 Oct 11;22(4):552-560.e5.)

8. Mouse Anti-Phosphotyrosine Antibody, clone 4G10 (Sigma Millipore 05-321) was validated and published by K Shuai., et al. (Science. 1993 Sep 24;261(5129):1744-6.)

9. Polyclonal rabbit Anti-Helicobacter pylori CGAT-specific antibody was created in this study and was validated by comparing samples that did not contain CGAT.

Eukaryotic cell lines

Policy information about cell lines

Cell line source(s)	Both AGS cell line and KATO III cell line were obtained from the American Type Culture Collection (ATCC)
Authentication	Both AGS and KATO III cell line were certified by ATCC. ATCC uses morphology, karyotyping, and PCR based approaches to confirm the identity of AGS cell line and KATO II cell line.
Mycoplasma contamination	Cell lines were tested negative for mycoplasma using PCR based approach.
Commonly misidentified lines (See ICLAC register)	These cell lines used are not listed in the database of commonly misidentified cell lines.

Flow Cytometry

Plots

Confirm that:

☒ The axis labels state the marker and fluorochrome used (e.g. CD4-FITC).

☒ The axis scales are clearly visible. Include numbers along axes only for bottom left plot of group (a 'group' is an analysis of identical markers).

☐ All plots are contour plots with outliers or pseudocolor plots.

☒ A numerical value for number of cells or percentage (with statistics) is provided.

Methodology

Sample preparation	Cells treated with compounds, enzymes, OMVs, blocking antibodies, or inhibitors were infected with H. pylori 26695 (MOI = 50) at 37 °C for 1 h. After the cells were washed to remove non-adherent bacteria, they were detached with 2 mM EDTA and fixed with 2% formaldehyde. Cells with plasma membrane-associated H. pylori bacteria were stained with rabbit anti-H. pylori antibody (abcam, 1:1,000) at 4 °C for 16 h and then washed with PBS. The secondary antibody was fluorescein isothiocyanate (FITC)-conjugated goat anti-rabbit antibody (Invitrogen, 1:1000), which was applied at 4 °C for 1 h, and the samples were then washed with PBS. Cells were analyzed by a flow cytometry system. Adherence was quantitated in terms of the proportion of cells with adherent H. pylori.
Instrument	BD FACSCalibur Calibur Flow Cytometer 4 Color was used for data collection.
Software	BD FACStation Software Version 3.3 was used for data acquisition.
Cell population abundance	At least over 10000 cells were collected
Gating strategy	For gating strategy, cell debris and bacteria without cell attachment were first removed by gating the main cell population using the FSC/SSC gating. Mock-treated cells (cells only without bacterial infection) that had been treated with the same staining procedure as aforementioned were used to distinguish the boundary between the positive or negative staining populations. An identical threshold was applied for all samples within the same cell line.

☒ Tick this box to confirm that a figure exemplifying the gating strategy is provided in the Supplementary Information.
